# Immunometabolic features of natural killer cells are associated with infection outcomes in critical illness

**DOI:** 10.3389/fimmu.2024.1334882

**Published:** 2024-02-15

**Authors:** Kuei-Pin Chung, Jia-Ying Su, Yi-Fu Wang, Bugi Ratno Budiarto, Yu-Chang Yeh, Jui-Chen Cheng, Li-Ta Keng, Yi-Jung Chen, Ya-Ting Lu, Yi-Hsiu Juan, Kiichi Nakahira, Sheng-Yuan Ruan, Jung-Yien Chien, Hou-Tai Chang, Jih-Shuin Jerng, Yen-Tsung Huang, Shih-Yu Chen, Chong-Jen Yu

**Affiliations:** ^1^ Department of Laboratory Medicine, National Taiwan University Hospital, Taipei, Taiwan; ^2^ Department of Laboratory Medicine, College of Medicine, National Taiwan University, Taipei, Taiwan; ^3^ Institute of Molecular Biology, Academia Sinica, Taipei, Taiwan; ^4^ Institute of Statistical Science, Academia Sinica, Taipei, Taiwan; ^5^ Institute of Biomedical Informatics, National Yang Ming Chiao Tung University, Taipei, Taiwan; ^6^ Bioinformatics Program, Taiwan International Graduate Program, Academia Sinica, Taipei, Taiwan; ^7^ Institute of Biomedical Sciences, Academia Sinica, Taipei, Taiwan; ^8^ Taiwan International Graduate Program in Molecular Medicine, National Yang Ming Chiao Tung University and Academia Sinica, Taipei, Taiwan; ^9^ Department of Anesthesiology, National Taiwan University Hospital, Taipei, Taiwan; ^10^ Department of Integrated Diagnostics & Therapeutics, National Taiwan University Hospital, Taipei, Taiwan; ^11^ Department of Internal Medicine, National Taiwan University Hospital, Hsinchu, Taiwan; ^12^ Department of Internal Medicine, National Taiwan University Hospital, Taipei, Taiwan; ^13^ Department of Pharmacology, Nara Medical University, Kashihara, Nara, Japan; ^14^ Department of Critical Care Medicine, Far Eastern Memorial Hospital, New Taipei, Taiwan; ^15^ Department of Industrial Engineering and Management, Yuan Ze University, Taoyuan, Taiwan; ^16^ Department of Internal Medicine, College of Medicine, National Taiwan University, Taipei, Taiwan

**Keywords:** chronic critical illness, nosocomial infection, natural killer cells, metabolism, NRF1, CPT1a

## Abstract

Immunosuppression increases the risk of nosocomial infection in patients with chronic critical illness. This exploratory study aimed to determine the immunometabolic signature associated with nosocomial infection during chronic critical illness. We prospectively recruited patients who were admitted to the respiratory care center and who had received mechanical ventilator support for more than 10 days in the intensive care unit. The study subjects were followed for the occurrence of nosocomial infection until 6 weeks after admission, hospital discharge, or death. The cytokine levels in the plasma samples were measured. Single-cell immunometabolic regulome profiling by mass cytometry, which analyzed 16 metabolic regulators in 21 immune subsets, was performed to identify immunometabolic features associated with the risk of nosocomial infection. During the study period, 37 patients were enrolled, and 16 patients (43.2%) developed nosocomial infection. Unsupervised immunologic clustering using multidimensional scaling and logistic regression analyses revealed that expression of nuclear respiratory factor 1 (NRF1) and carnitine palmitoyltransferase 1a (CPT1a), key regulators of mitochondrial biogenesis and fatty acid transport, respectively, in natural killer (NK) cells was significantly associated with nosocomial infection. Downregulated NRF1 and upregulated CPT1a were found in all subsets of NK cells from patients who developed a nosocomial infection. The risk of nosocomial infection is significantly correlated with the predictive score developed by selecting NK cell-specific features using an elastic net algorithm. Findings were further examined in an independent cohort of COVID-19-infected patients, and the results confirm that COVID-19-related mortality is significantly associated with mitochondria biogenesis and fatty acid oxidation pathways in NK cells. In conclusion, this study uncovers that NK cell-specific immunometabolic features are significantly associated with the occurrence and fatal outcomes of infection in critically ill population, and provides mechanistic insights into NK cell-specific immunity against microbial invasion in critical illness.

## Introduction

Advances in critical care have decreased the mortality caused by acute critical illness, however, patients who survive the early stages of critical illness may fail to recover and can develop chronic critical illness (CCI) (1). Epidemiological studies have demonstrated that the transition from acute to chronic critical illness occurs after 10 days in intensive care units (2) and revealed an association of CCI with high mortality and a massive economic burden on the medical care system (3, 4). Immune dysfunction in CCI is associated with an increased risk of nosocomial infection (5), which leads to increased healthcare costs and mortality (6, 7). Although sepsis, which is an important risk factor for CCI (2, 3, 8, 9), may contribute to immune dysfunction in CCI, characterized by elevated levels of pro-inflammatory and anti-inflammatory cytokines and altered levels of circulating immune cell populations (10–16), the exact nature of the immune dysfunction associated with CCI is not fully understood. Restoring immune dysfunction in CCI may prevent nosocomial infection and promote recovery from CCI.

Immunometabolism is the intricate relationship between cellular metabolism and immune cell function. Metabolic pathways, such as glycolysis, oxidative phosphorylation, fatty acid oxidation, and amino acid metabolism, play critical roles in shaping immune cell activation, differentiation, and effector functions (17). Dysregulation of immunometabolism has been implicated in various immune-related diseases including autoimmune disorders, cancer, infectious diseases, and chronic inflammation (18). A recent study demonstrated that sepsis-related intrinsic metabolic defects in monocytes cause immunosuppression and increased mortality in a murine model (19). However, it is unclear whether immunometabolic dysregulation leads to immune dysfunction and increased susceptibility to nosocomial infection in patients with CCI. Furthermore, although immune dysfunction associated with nosocomial infection has been explored in several studies by analyzing the abundance and function of immune cells in peripheral blood mononuclear cells (PBMCs) (20–22), the metabolic activities in immune cells had only been assessed in bulk by techniques such as extracellular flux analyses (23, 24).

Studying immunometabolism at the single-cell level is crucial for unraveling the complex mechanisms underlying immune cell function. It is difficult to assess metabolic activities in various immune cell types simultaneously using platforms such as extracellular flux analyses (24). Mass cytometry, also known as cytometry by time-of-flight (CyTOF), offers a powerful approach for high-dimensional single-cell analysis, enabling simultaneous measurement of multiple markers and elucidation of cellular heterogeneity. Single-cell immunometabolic regulomic profiling (scMEP), which employs mass cytometry, has been used to characterize cell identities and metabolic features at single-cell resolution (23, 25), enabling exploration of the immunometabolic alternations associated with various diseases.

In this prospective exploratory study, we applied scMEP to characterize the metabolic regulators of various immune cells in PBMCs to identify the immunometabolic features associated with the risk of nosocomial infection in patients with CCI. The findings highlight the significant association between natural killer (NK) cell-specific immunometabolic features involving mitochondrial biogenesis and fatty acid β-oxidation and the risk of nosocomial infection in CCI. Through exploratory analyses using single-cell RNA sequencing (scRNA-seq) datasets from COVID-19-infected subjects, we found the identified NK cell-specific immunometabolic features are significantly correlated with host survival in COVID-19 infection. Our study thus reveals the clinical outcomes related to infection in critical ill population are significantly associated with NK cell-specific immunometabolism, and sheds light on NK cell-specific immunity protective against infection in critical illness.

## Materials and methods

### Study population

This prospective observational study was conducted at the respiratory care center (RCC) at the National Taiwan University Hospital (NTUH), a specialized step-down protocol-driven weaning facility, and included patients with CCI who received mechanical ventilator support for more than 10 days (26). The Institutional Review Board of NTUH approved the study protocol (201303047RINC). The study subjects, enrolled from August 2013 through March 2015, included adult patients (≥ 20 years) who were transferred from intensive care units (ICUs) to the RCC for weaning and who did not have evidence of active infection, did not receive antibiotics or did not receive antibiotics for more than 3 days prior to admission to the RCC, and did not have fever or hypothermia for more than 24 h prior to admission to the RCC. Patients who had systemic autoimmune diseases, hematological malignancies, advanced malignancy with inevitable short-term mortality, human immunodeficiency virus infection, patients receiving immunosuppressive treatment, or patients who refused to consent were excluded from the study.

### Clinical data and outcomes

Data on the demographics, comorbidities, and laboratory examination results at admission to the RCC were collected. The occurrence of sepsis, septic shock, and acute respiratory distress syndrome (ARDS) at ICU admission (27, 28), nosocomial infection during ICU stay, and ventilator-dependent days before admission to RCC were recorded. Nosocomial infection was defined according to the 2014 surveillance criteria of the Centers for Disease Control and Prevention’s National Healthcare Safety Network. All study subjects were followed for the occurrence of nosocomial infection for 6 weeks after RCC admission, hospital discharge, or death, whichever occurred earlier. Nosocomial infection was determined by attending physicians and was independently reviewed and confirmed by L.T.K. and K.P.C. Adverse clinical outcomes were assessed in the study subjects who died during their stay at the RCC and in those who were readmitted to ICUs due to deterioration in clinical condition.

### Sample collection and processing

We collected 10 mL of whole blood in an ethylenediaminetetraacetic acid-coated tube from participants who gave informed consent upon admission to the RCC. Whole blood samples were centrifuged at 800 g for 10 minutes at 4°C, and the plasma was transferred to a 15-mL polypropylene tube. The buffy coat was used to isolate PBMCs. The plasma was further centrifuged at 2000 g for 10 minutes at 4°C, and was aliquoted and stored at -80°C before cytokine measurements. PBMCs were isolated using the Ficoll-paque gradient (GE Healthcare) and were subsequently aliquoted into fetal bovine serum (FBS; Biological Industries)-enriched freezing medium containing 10% dimethyl sulfoxide, and stored in liquid nitrogen. Each cryotube contained 2~4 x 10^6^ PBMCs.

### Cytokine measurements

The plasma levels of cytokines, including tumor necrosis factor (TNF)-α, interleukin (IL)-6, IL-8, IL-10, and IL-15, were measured using a multiplex cytokine kit (MILLIPLEX MAP Human Cytokine/Chemokine Panel, Millipore Corporation) according to manufacturer’s instructions. The cytokine level was designated as 0 pg/mL when the concentration was below the detection limit.

### Mass cytometry analysis

PBMCs were washed once using serum-free Roswell Park Memorial Institute (RPMI) 1640 medium. Cells were then stained with cisplatin (Sigma-Aldrich) at a final concentration of 25 μM for 1 min at room temperature to label dead cells and then quenched by equal volume of RPMI1640 medium with 10% FBS for viability staining. Next, the cells were fixed with 1.6% paraformaldehyde (Electron Microscopy Sciences) in serum-free RPMI1640 at room temperature for 10 min. PBMCs from different donors were barcoded with Cell-ID 20-Plex Pd Barcoding Kit (Fluidigm), pooled and stained for 26 lineage markers and 16 metabolic regulators (**Table 1**). For surface marker staining, cells were incubated with a cell-surface antibody cocktail prepared in cell staining media (CSM), containing 1x phosphate-buffered saline (PBS), 0.5% protease-free bovine serum albumin, and 0.02% NaN3, in a final volume of 100 μL for 1 hour at room temperature. After washing once with CSM, cells were permeabilized with 100% ice-cold methanol for 10 minutes. For intracellular marker staining, cells were washed twice with CSM and stained with an intracellular antibody cocktail prepared in CSM in a final volume 100 μL for 1 hour at room temperature. After staining, cells were washed twice with CSM, and then stained with Cell-ID Intercalator-Ir (191Ir and 193Ir; Fluidigm) at a final concentration of 125 nM in 1000 μL 1.5% fresh paraformaldehyde (diluted in 1xPBS) overnight at 4°C for DNA staining. Finally, cells were resuspended in MilliQ water containing EQ™ Four Element Calibration Beads (Fluidigm) for normalization. Data were acquired using a CyTOF2 mass cytometer (Fluidigm). Data in raw flow cytometry standard files were normalized and debarcoded using the Premessa R package (http://github.com/ParkerICI/premessa). To eliminate batch variation, data were aligned and corrected using the Spectre package (https://github.com/ImmuneDynamics/Spectre). The data were uploaded and gated in Cytobank, and marker intensities were arcsinh-transformed with a cofactor of 5 before analyses.

**Table 1:** Antibodies used for mass cytometry analysis.

Target	Metal	Element	Clone	Vendor
Lineage markers				
CD1c	172	Yb	L161	Biolegend
CD3	113	In	UCHT1	Invitrogen
CD4	145	Nd	RPA-T4	Fluidigm
CD8	146	Nd	RPA-T8	Fluidigm
CD11c	140	Ce	Bu15	Biolegend
CD14	160	Gd	M5E2	Biolegend
CD16	165	Ho	3G8	Fluidigm
CD19	142	Nd	HIB19	Fluidigm
CD38	163	Dy	HIT2	Biolegend
CD39	147	Sm	A1	Biolegend
CD45	89	Y	HI30	Fluidigm
CD45RA	153	Eu	HI100	Fluidigm
CD56	176	Yb	NCAM16.2	BD Bioscience
CD57	139	La	HCD57	Biolegend
CD66b	141	Pr	G10F5	Biolegend
CD86	156	Gd	IT2.2	Fluidigm
CD123	144	Nd	6H6	Biolegend
CD141	149	Sm	1A4	BD
CD161	158	Gd	HP-3G10	Biolegend
CD197/CCR7	159	Tb	G043H7	Fluidigm
CCR2	170	Er	48607	R&D
FoxP3	162	Dy	PCH101	Fluidigm
HLA-DR	174	Yb	L243	Fluidigm
PD1	175	Lu	EH12.2H7	Fluidigm
TCRVα7.2	166	Er	3C10	Biolegend
TCRgd	173	Yb	331202	Biolegend
Metabolic regulators				
ATP5a	115	In	7H10BD4F9	Abcam
ACADM	171	Yb	3B7BH7	Abcam
CPT1	154	Sm	8F6AE9	Abcam
CS	152	Sm	EPR8067	Abcam
Cytc	150	Nd	6H2.B4	Biolegend
DRP1	148	Nd	EPR19274	Abcam
GAPDH	155	Gd	6C5	Invitrogen
GLUT1	209	Bi	EPR3915	Abcam
HADHA	143	Nd	EPR17940	Abcam
HK2	168	Er	3D3	Abcam
LDH	167	Er	EP1566Y	Abcam
NRF1	157	Gd	EPR5554	Abcam
OGDH	164	Dy	poly-clone	Invitrogen
OPA1	169	Tm	1E81D9	Abcam
PGC1α	151	Eu	4A8	Abcam
VDAC	161	Dy	20B12AF2	Abcam

CD, cluster of differentiation; CCR, CC chemokine receptor; FoxP3, forkhead box P3; HLA-DR, human leukocyte antigen-DR; PD1, programmed death-1; TCRVα7.2, T cell receptor Vα7.2; TCRgd, T cell receptor γ/δ; ATP5a, ATP synthase F1 subunit alpha; ACADM, acyl-CoA dehydrogenase medium chain; CPT1, carnitine pamitoyltransferase 1; CS, citrate synthase; CytC, cytochrome C; DRP1, dynamin-related protein 1; GAPDH, glyceraldehyde-3-phosphate dehydrogenase; GLUT1, glucose transporter 1; HADHA, hydroxyacyl-CoA dehydrogenase trifunctional multi-enzyme complex subunit α; HK2, hexokinase 2; LDH, lactate dehydrogenase; NRF1, nuclear respiratory factor 1; OGDH, oxoglutarate dehydrogenase; OPA1, optic atrophy type 1; PGC1α, peroxisome proliferator-activated receptor γ coactivator 1α; VDAC, voltage-dependent anion channel.

### Statistical analyses

Data are presented as medians [interquartile ranges], mean ± standard deviation or number (percentage). For continuous variables, the Mann-Whitney U test was used to compare differences between the two groups. Discrete variables were compared using Pearson’s χ^2^ test or Fisher’s exact test as appropriate. For mass cytometric data, immune features, including cell abundance and expression levels of specific markers, were calculated for each immune subset. Uniform manifold approximation and projection (UMAP) was performed using Cytobank with 23 immune cell lineage markers (CCR2, CD11c CD123, CD14, CD141, CD16, CD161, CD197, CD1c, CD3, CD38, CD39, CD4, CD45RA, CD56, CD57, CD8, CD86, Foxp3, HLA-DR, PD-1 TCRgd, TCRVa7.2). The parameters were equal events numbers, number of neighbors = 15, and minimum distance = 0.01. NK population was determined by high CD56 cluster according to UMAP results. Secondary UMAP was performed with 16 metabolic regulators (**Table 1**). The parameters were equal events numbers, number of neighbors = 30, and minimum distance = 0.01. FlowSOM analyses were performed by Cytobank with 10 metaclusters. Unsupervised clustering of patient data was performed using multidimensional scaling (MDS) based on the distance matrix of pairwise squared ranking differences between immunological features including mass cytometric data and plasma cytokine levels (29). The raw data were first normalized by Z transformation with means and standard deviations of the respective variables. To determine the appropriate dimensionality of MDS, we used the “elbow” method by plotting the number of dimensions versus the values of a loss function. The loss function is called “stress” and is given by:


$$Stress=\sqrt{\frac{\sum \;{\left(d_{ij}-{\hat{d}}_{ij}\right)}^{2}}{\sum \;d_{ij}^{2}}},$$

where ${\hat{d}}_{ij}$ and $d_{ij}$ are the predicted distance by MDS and the estimated distance from the distance matrix between patient i and j . Area under the receiver operating characteristic curve (AUROC) was utilized to assess the performance of classification regarding nosocomial infection for each coordinate of MDS and was calculated using the R package “precrec” (30). The 95% confidence interval (CI) of AUROC was obtained using bootstrapping with 1,000 replications. The Spearman correlation coefficient was calculated to evaluate the importance of each feature for each MDS coordinate, and the *p* values for testing correlation coefficients were adjusted for multiplicity using the R package “qvalue” (31). The elastic net logistic regression was employed to conduct variable selection among the significant immunologic features using the R package “glmnet” to identify the key attributes associated with nosocomial infection (32). Based on the variables selected by the elastic net algorithm, a predicted score was derived using the equation below:


$$Predicted\;score=\frac{e^{\left(\alpha +\sum_{k}\;\beta_{k}V_{k}\right)}}{\left(1+e^{\left(\alpha +\sum_{k}\;\beta_{k}V_{k}\right)}\right)},$$

where α is the intercept and $\beta_{k}$ is the estimated regression coefficient from the elastic net regression for the feature $V_{k}$ . Mann–Whitney U tests were used to evaluate the differences of the predicted scores between subgroups dichotomized based on nosocomial infection occurrence after RCC admission. The performances of the model were evaluated by calculating AUROC, as mentioned above. Logistic regression analyses were used to calculate the unadjusted and adjusted odds ratios of nosocomial infection for the predicted score based on immunometabolic features. The arcsinh mean value of metabolic regulator expression in each immune subset was assessed and visualized through a heatmap. The heatmap plot was generated using the pheatmap R package (https://github.com/raivokolde/pheatmap).

Regarding scRNA-seq analysis, The datasets, GSE145926 (33) and GSE157344 (34) were downloaded from Gene Expression Omnibus and were utilized to examine bronchoalveolar fluid cell samples obtained from moderate (patients requiring oxygen without respiratory support), severe (patients requiring admission to ICU and/or non-invasive/mechanical ventilation), and deceased COVID-19-infected patients. In order to comprehensively cover the entire spectrum of disease severity, we opted to merge two cohorts and employ computational integration techniques to mitigate potential batch effects. The rationale behind our sample selection aims to achieve a balance in age-matched samples from both cohorts, aligning with established practices in meta-analysis studies (35). The selected dataset composition was as follows: three datasets for the moderate group, six datasets for the severe group, and five datasets for the deceased group. Independent validation for the results from the two datasets above (GSE145926 and GSE157344) was done through analyzing GSE161918 dataset, which contains CITE sequencing-based cell annotation (36). For analyzing GSE145926 and GSE157344 datasets, the Seurat package (version 4.0.4, https://github.com/satijalab/seurat) in R (version 4.0.5) was employed. Cells with RNA feature counts ranging from 200 to 7500 and mitochondrial content less than 5% were retained for further analysis. Data scaling and transformation were performed by applying the ‘NormalizeData’ function, which scaled the data by a factor of 10,000 and transformed it into natural-log transformed values, where each cell’s value was divided by the total counts for that cell and multiplied by the scale factor. For batch corrections, ‘FindIntegrationAnchors’ and ‘IntegrateData’ functions were used for anchor-based integration, relying on matched biological states or ‘anchors’ to identify cells across different datasets. Dimensionality reduction was achieved by applying the ‘RunPCA’ function to the integrated Seurat object, using the first 30 principal components. The high-dimensional cellular data were visualized using the t-distributed stochastic neighbor embedding (tSNE) method. A shared nearest-neighbor graph was constructed using the first 30 principal components with the ‘FindNeighbors’ function, and a graph-based modularity-optimization algorithm, specifically the Louvain method, was employed for community detection via ‘FindClusters’. Differentially expressed genes were identified using the default ‘FindMarkers’ function in Seurat, which relies on Mann-Whitney U tests. The cell identity of each cluster was automatically defined using the SingleR package (https://github.com/dviraran/SingleR) within the R environment. For analysis using GSE161918 dataset, normalized seurat object generated by original Author was used. Dimensionality reduction was achieved by applying the ‘RunPCA’ function to the normalized Seurat object, using the first 30 principal components. The high-dimensional cellular data were visualized using the tSNE method. A shared nearest-neighbor graph was constructed using the first 15 principal components with the ‘FindNeighbors’ function, and a graph-based modularity-optimization algorithm, specifically the Louvain method, was employed for community detection via ‘FindClusters’. T cells clusters were anchored to CITE-sequencing-based cell id provided in metadata to preserve the cell type annotation similar the original paper. Lastly, gene signatures related to mitochondrial fatty acid oxidation (GO:0031998) and mitochondrial biogenesis (R-HSA-1592230) were incorporated into the Seurat object using the ‘addmodule score’ function to assess NK cells in each clinical condition. Kruskal-Wallis tests were applied for multiple group comparison of the scores, and the *p* values were adjusted using the Dunn’s method.

Statistical significance was defined as a two-sided *p*-value of < 0.05. Statistical analyses and figure plotting were performed using SPSS (version 17.0; IBM Corporation), GraphPad Prism (version 9.4.0; GraphPad Software), or R 4.2.0.

## Results

### Clinical features of the study population

During the study period, 37 patients with CCI who were admitted to the RCC were recruited; their clinical characteristics are described in **Table 2**. Sixteen patients (43.2%) developed nosocomial infection after admission to the RCC, and pneumonia (11 of 16, 68.8%) was the most common diagnosis. Demographic features, co-morbidities other than congestive heart failure, and laboratory examination results were not significantly associated with the occurrence of nosocomial infection in CCI. Patients who developed nosocomial infection had significantly increased risk of worse clinical outcomes compared to those who did not (*p* = 0.012 by Fisher’s exact test).

**Table 2:** Clinical characteristics of patients upon admission to the respiratory care center (RCC).

Parameters	Entire population	Nosocomial infection after RCC admission		
		No	Yes^a^	*p* value^b^
**Number**	37	21	16	
**Age**	79.0 [14.0]	82.0 [8.0]	77.0 [23.0]	0.094
**Gender**				0.368
Male	20 (54.1)	10 (47.6)	10 (62.5)	
Female	17 (45.9)	11 (52.4)	6 (37.5)	
Co-morbidities				
CHF	6 (16.2)	6 (28.6)	0 (0.0)	0.027
CAD	11 (29.7)	6 (28.6)	5 (31.3)	1.000
DM	14 (37.8)	7 (33.3)	7 (43.8)	0.517
Hypertension	24 (64.9)	14 (66.7)	10 (62.5)	0.793
CKD	17 (45.9)	10 (47.6)	7 (43.8)	0.815
Neurologic diseases	17 (45.9)	12 (57.1)	5 (31.3)	0.117
Malignancy	14 (37.8)	7 (33.3)	7 (43.8)	0.517
Laboratory results				
Leukocyte (x10^3^/μL)	8.4 [4.3]	7.6 [4.4]	10.3 [7.6]	0.158
Platelet (10^3^/μL)	190.0 [119.0]	186.0 [168.0]	210.0 [112.5]	0.724
Hemoglobin (g/dL)	9.2 [1.6]	9.7 [1.2]	8.9 [1.5]	0.133
Total bilirubin (mg/dL)	0.6 [0.5]	0.5 [0.3]	0.6 [0.5]	0.434
Creatinine (mg/dL)	1.0 [1.9]	0.9 [1.9]	1.3 [2.6]	0.713
**Worse outcomes**	8 (21.6)	1 (4.8)	7 (43.8)	0.012
ICU readmission	7	0 (0.0)	7 (43.8)	
Death	1	1 (4.8)	0 (0.0)	

Data are presented as medians [interquartile ranges] or numbers (percentages). CHF, congestive heart failure; CAD, coronary arterial disease; DM, diabetes mellitus; CKD, chronic kidney disease; ICU, intensive care unit.

a Including pneumonia (n=11), intra-abdominal infection (n=3), primary bloodstream infection (n=1), and brain abscess (n=1).

b Discrete variables were compared using Pearson’s χ2 test or Fisher’s exact test, as appropriate, while continuous variables were compared using Mann-Whitney U test.

### Unsupervised immunometabolic clustering reveals the link between NK cell-specific features and nosocomial infection risk

**Figure 1:**
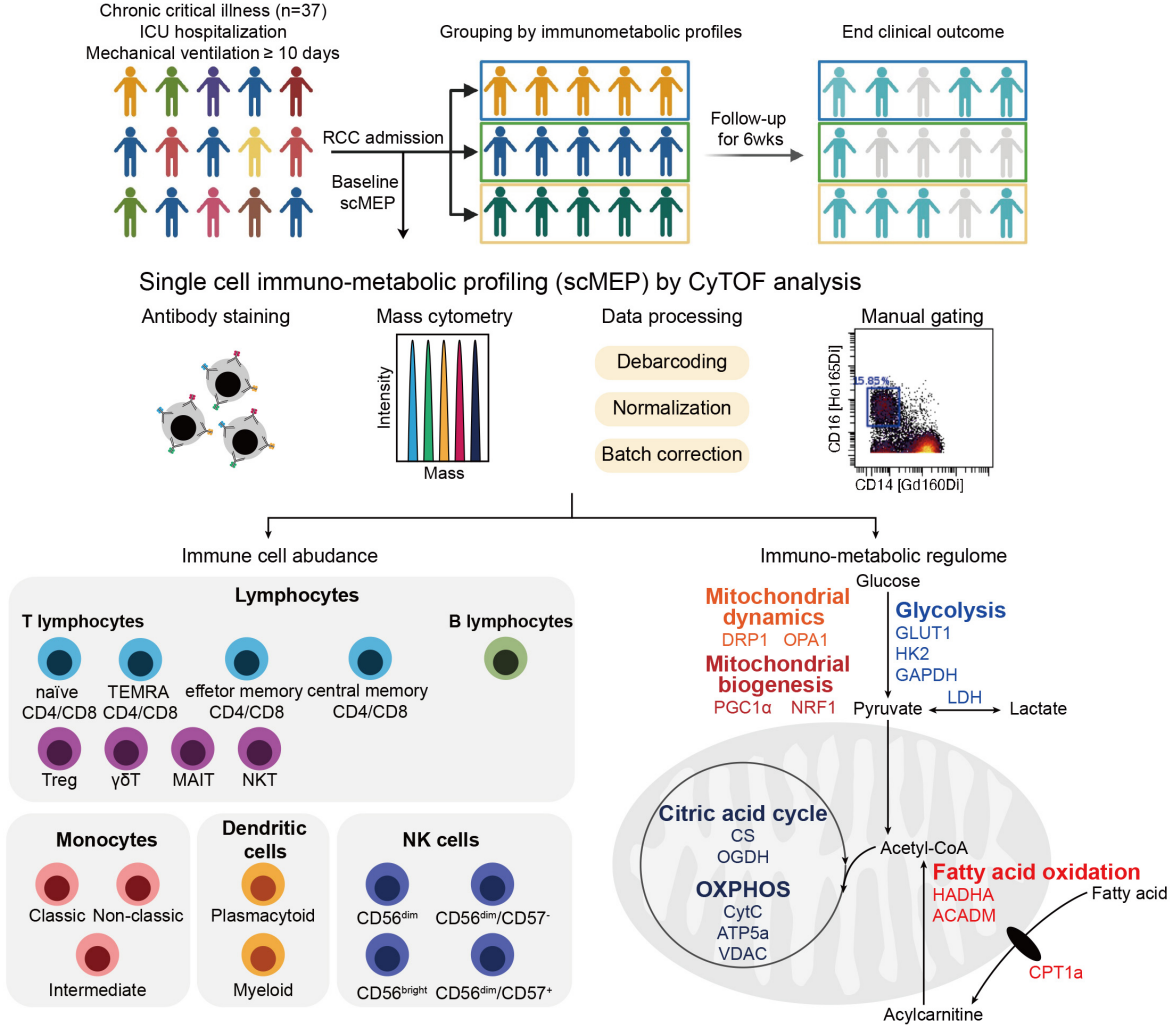
Overview of study and the single-cell immunometabolic regulomic profiling (scMEP) process. Patients with chronic critical illness, defined as intensive care unit (ICU) hospitalization with mechanical ventilation support for more than 10 days, are followed to determine the clinical outcome, the occurrence of nosocomial infection. Samples are analyzed through scMEP, which employs cytometry by time of flight (CyTOF), automated data processing, and manual gating to determine immune cell abundances and to evaluate the immunometabolic regulome. (TEMRA CD4/CD8, terminally differentiated effector memory CD4+/CD8+ T lymphocyte; Treg, regulatory T lymphocyte; γδT, γδT lymphocyte; MAIT, mucosal-associated invariant T lymphocyte; NKT, natural killer T lymphocyte; NK, natural killer cells; GLUT1, glucose transporter 1; HK2, hexokinase 2; GAPDH, glyceraldehyde-3-phosphate dehydrogenase; LDH, lactate dehydrogenase; HADHA, hydroxyacyl-CoA dehydrogenase trifunctional multi-enzyme complex subunit α; ACADM, acyl-CoA dehydrogenase medium chain; CPT1a, carnitine pamitoyltransferase 1a; DRP1, dynamin-related protein 1; OPA1, optic atrophy type 1; PGC1α, peroxisome proliferator-activated receptor γ coactivator 1α; NRF1, nuclear respiratory factor 1; CS, citrate synthase; OGDH, oxoglutarate dehydrogenase; OXPHOS, oxidative phosphorylation; CytC, cytochrome C; ATP5a, ATP synthase F1 subunit alpha; VDAC, voltage-dependent anion channel).

**Figure 2:**
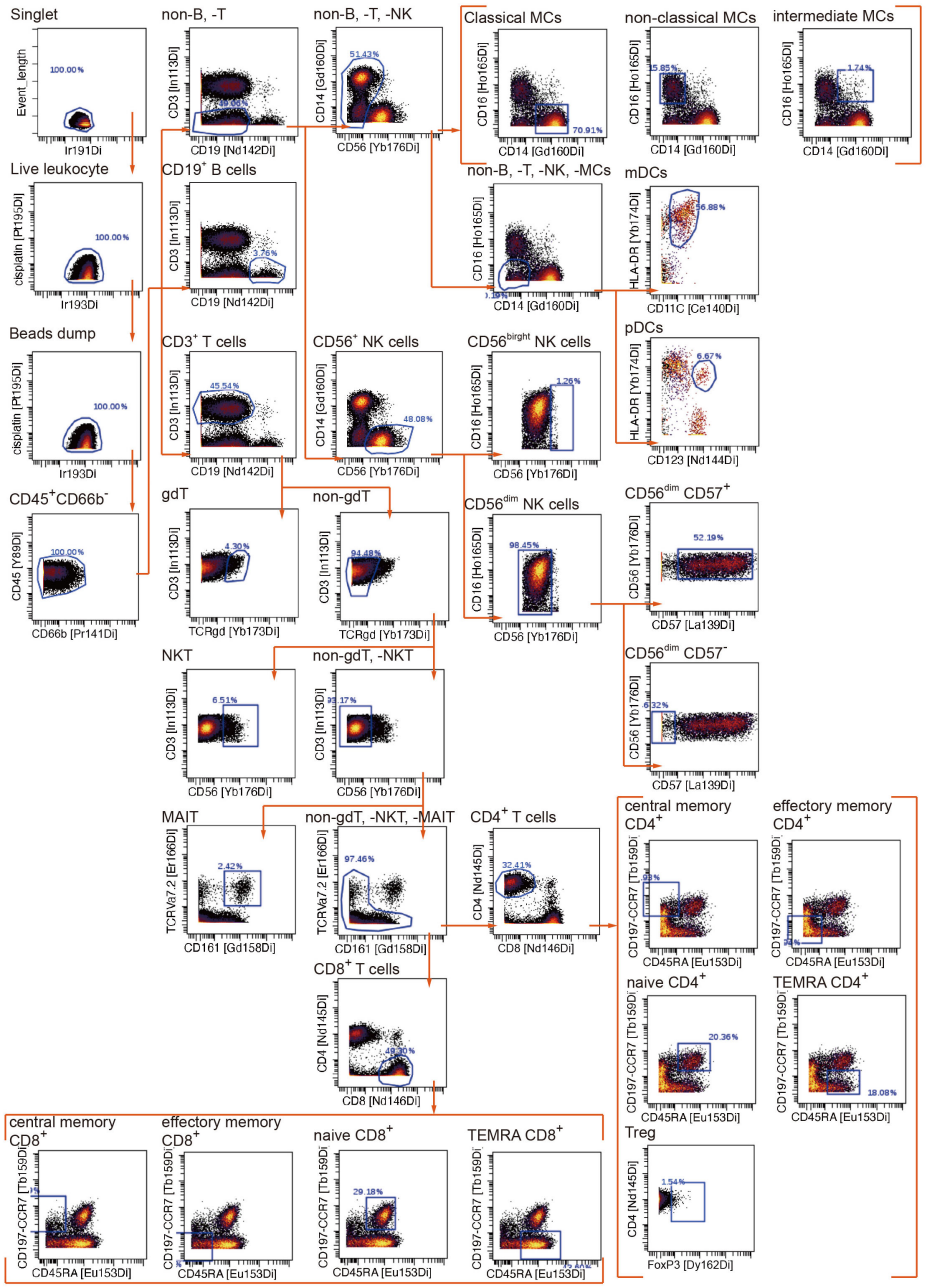
Manual gating algorithm to identify various immune cell subsets by mass cytometric analyses.

**Figure 3:**
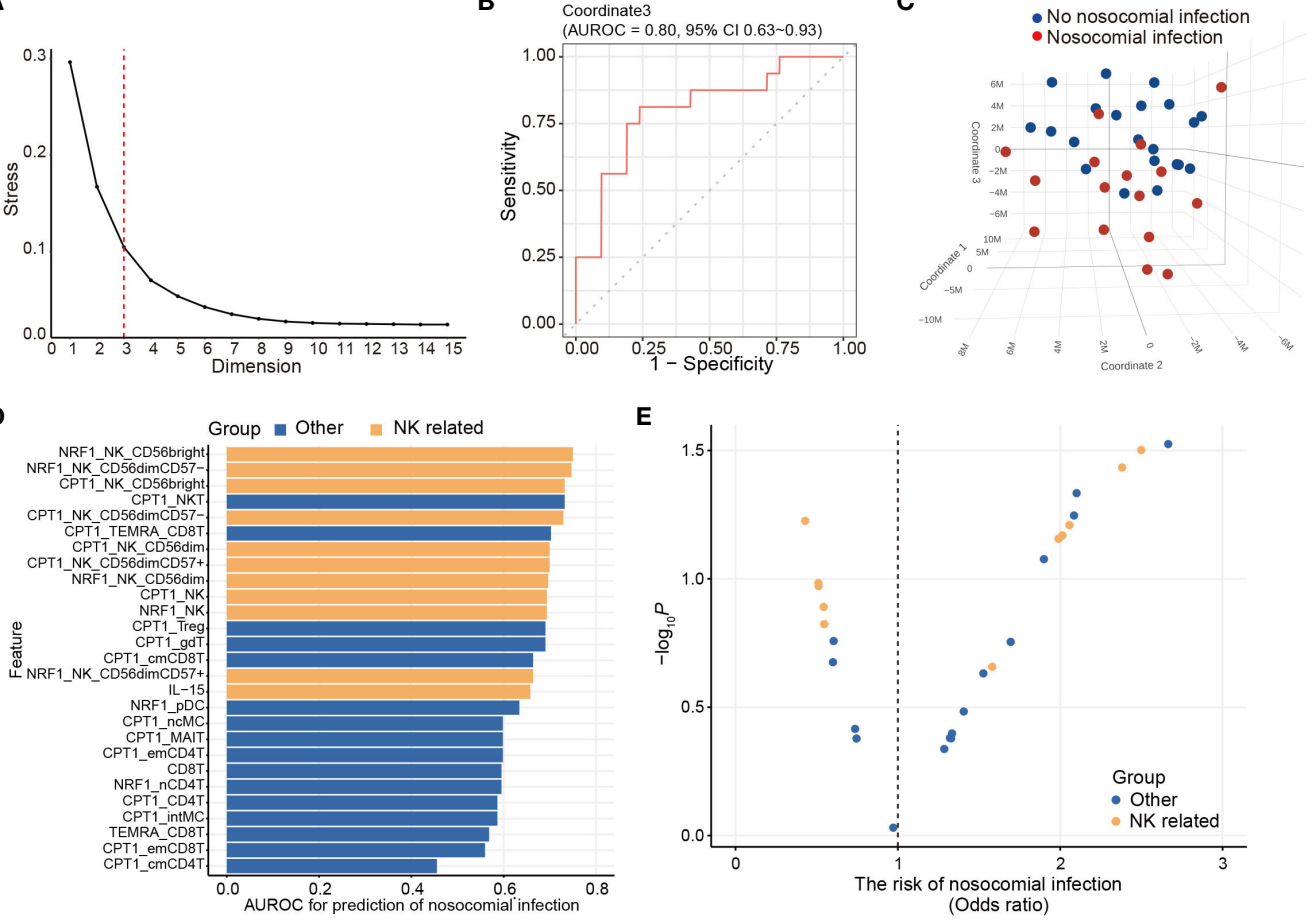
Unsupervised immunologic clustering of the study population. **(A)** Stress versus dimension plot is generated to evaluate the ideal number of the dimensions for multidimensional scaling (MDS) analysis. **(B)** Area under receiver operating characteristics curve (AUROC) and associated 95% confidence interval are calculated for assessing the performance of MDS coordinate 3 in predicting the risk of nosocomial infection. **(C)** Three-dimensional MDS plot is generated to visualize the clustering of the study population based on the development of nosocomial infection. **(D, E)** Twenty-seven immunometabolic features are significantly correlated with coordinate 3 of the MDS plot (also see **Table 3**). AUROC **(D)** and odds ratio **(E)** of nosocomial infection are calculated for these 23 features. The odds ratio and the *p* value are determined by logistic regression analyses.

To uncover the immunometabolic features associated with nosocomial infection in CCI, we used scMEP to quantify proteins that regulate metabolic pathway activity across different immune subsets in samples collected from subjects upon admission to RCC (**Figure 1**). Using immunophenotypic markers, 21 major immune subsets were manually gated (**Figure 2**). The abundance and the metabolic protein expression profiles of each immune subset were quantified, resulting in 357 immune features for each patient in the study cohort. In addition, the circulating levels of five cytokines, IL-10, IL-15, IL-6, IL-8, and TNF alpha, were measured for each patient, and the results showed that patients with nosocomial infection had significantly increased IL-10 levels at RCC admission (**Supplementary Figure 1A**). To identify the specific immune features associated with nosocomial infection, we first performed MDS analysis to assess the degree of similarity among patients. In MDS analysis, stress is a quantitative measure of the dissimilarity between the reduced-dimensional representation and the original data. This measure guides determination of the number of dimensions that will retaining as much information as possible. Based on the relationship between stress and the number of dimensions (**Figure 3A**), we found that study subjects could be appropriately categorized using a three-dimensional MDS plot.

In order to determine their effectiveness in distinguishing between patients with nosocomial infection and those without, we assessed the performance of each coordinate using AUROC. Notably, only coordinate 3 exhibited statistically significant correlations, with AUROC value of 0.80 (95% CI of 0.63-0.93). The AUROC value for coordinate 1 was 0.58 (95% CI of 0.38-0.76) and that for coordinate 2 was 0.61 (95% CI of 0.41-0.79) (**Figure 3B**). Patients belonging to the nosocomial infection and non-nosocomial infection groups were visually separated from each other based on coordinate 3 (**Figure 3C**), indicating that the risk of nosocomial infection is significantly associated with the immunological features that correlated with coordinate 3 of the MDS plot. Twenty-seven immunological features were significantly correlation with coordinate 3 (**Table 3**). The correlations between these identified immunological features and the risk of nosocomial infection were evaluated by both AUROC (**Figure 3D**) and univariate logistic regression analyses (**Figure 3E**). To show the complete picture for the correlation between the immunometabolic features and the risk of nosocomial infection, we calculated and presented the extent of differential expression for all metabolic regulator in each immune subset, comparing patient subgroup with and without nosocomial infection (**Supplementary Figure 1B**). The findings consistently showed that the risk of nosocomial infection is associated with increased CPT1a in all NK cell subsets, NKT cells, and some T lymphocyte subsets. Collectively, our results revealed that NK cells were the predominant immune cell type, and CPT1a and NRF1 were the principal metabolic regulators associated with the risk of nosocomial infection.

**Table 3:** Correlation of immunometabolic features with coordinate 3 of the multidimensional scaling for unsupervised clustering^a^.

Immunometabolic features	Spearman correlation coefficient ρ	*p* value
NK cell related		
CPT1a_NK(CD56dim)	-0.7624	<0.001
CPT1a_NK	-0.7596	<0.001
CPT1a_NK(CD56dimCD57-)	-0.7525	<0.001
CPT1a_NK(CD56dimCD57+)	-0.7499	<0.001
CPT1a_NK(CD56bright)	-0.5446	<0.001
IL-15	-0.5294	0.001
NRF1_NK(CD56dimCD57-)	0.5439	0.001
NRF1_NK(CD56dim)	0.5503	<0.001
NRF1_NK(CD56bright)	0.5548	<0.001
NRF1_NK	0.5552	<0.001
NRF1_NK(CD56dimCD57+)	0.5690	<0.001
Others		
CPT1a_non-classic monocytes	-0.6854	<0.001
CPT1a_TEMRA CD8T	-0.6769	<0.001
CPT1a_Treg	-0.6399	<0.001
CPT1a_effecotr memory CD4T	-0.6375	<0.001
CPT1a_NKT	-0.6332	<0.001
CPT1a_central memory CD8T	-0.5907	<0.001
CPT1a_γδT	-0.5631	<0.001
CPT1a_CD4T	-0.5545	<0.001
CPT1a_effector memory CD8T	-0.5493	<0.001
CPT1a_MAIT	-0.5230	0.001
CPT1a_central memory CD4T	-0.4957	0.002
CPT1a_intermediate monocyte	-0.4780	0.003
NRF1_plasmacytoid dendritic cell	0.4749	0.003
NRF1_naïve CD4T	0.4908	0.002
CD8T (abundance)	0.4945	0.002
TEMRA CD8T (abundance)	0.5202	0.001

CD4T, CD4^+^ T lymphocytes; CD8T, CD8^+^ T lymphocytes; Treg, regulatory T lymphocytes; γδT, γδ T lymphocytes; TEMRA, terminally differentiated effector memory; NKT, natural killer T lymphocyte; NK, natural killer cells; MAIT, mucosal associated invariant T lymphocytes; CPT1a, carnitine pamitoyltransferase 1a; GLUT1, glucose transporter 1; NRF1, nuclear respiratory factor 1.

a Please refer to **Figure 3B** for the multidimensional scaling plot.

### Altered expression of NRF1 and CPT1a in NK cells is associated with an increased risk of nosocomial infection

**Figure 4:**
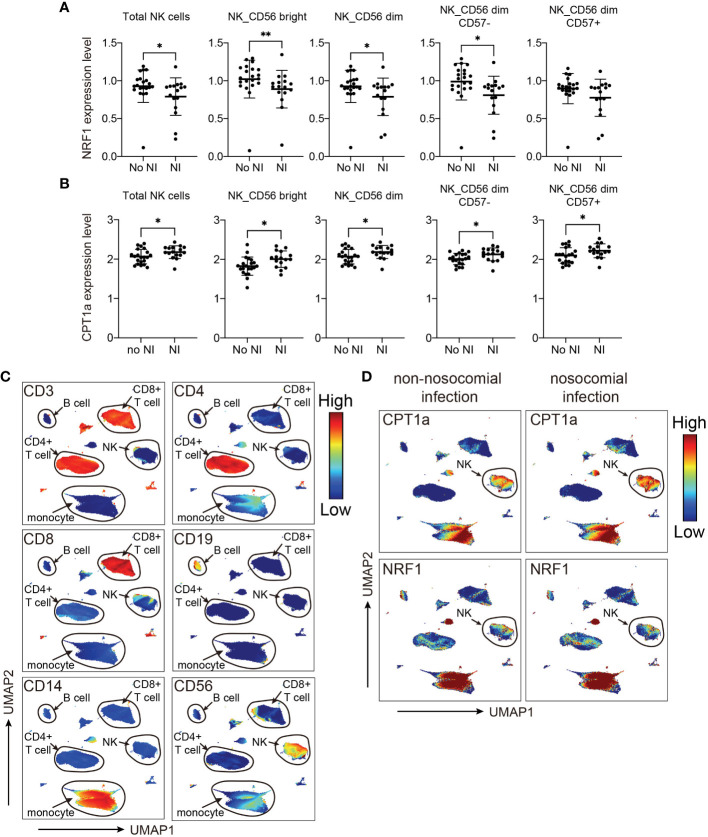
Levels of NRF1 and CPT1a expression in NK cells are correlated with occurrence of nosocomial infection (NI). **(A, B)** Plots of NRF1 **(A)**, and CPT1a **(B)** levels in indicated NK cell populations. The lines indicated mean ± standard deviation, and the *p* values are determined by Mann-Whitney U tests (** *p*< 0.01, * *p*< 0.05). **(C, D)** Uniform manifold approximation and projection (UMAP) plots visualizing indicated marker expression in all cells from patients with and those without NI. The major cell groups are annotated **(C)**, and the expression levels of **(D)** CPT1a and NRF1 in different immune cell subsets are demonstrated.

Based on the results from MDS analyses, we focused the analyses evaluating the association between nosocomial infection occurrence and NK cell-specific CPT1a and NRF1 expression. Notably, downregulation of intracellular NRF1 in the NK cell population and subpopulations was significantly associated with an increased risk of nosocomial infection, whereas CPT1a was upregulated in all NK cell subpopulations from patients who developed nosocomial infection (**Figures 4A–D**; **Supplementary Figure 2**). Although the transcriptional regulation activity of NRF1 is regulated by PGC1α, the levels of PGC1α in NK cells are not correlated with the risk of nosocomial infection (**Supplementary Figure 3**). To further investigate whether NK subsets with specific metabolic features are correlated with the risk of nosocomial infection, we applied uniform manifold approximation and projection (UMAP) analysis to compare the metabolic differences between patients with or without nosocomial infection (**Supplementary Figures 4A, B**). Compared to subjects without nosocomial infection, those with nosocomial infection exhibited an increased abundance of a specific cluster (**Supplementary Figures 4C, D**) which is characterized by elevated CPT1a expression and reduced NRF1 expression (**Supplementary Figures 4E, 5**). Collectively, the findings indicate that the expression NRF1 and CPT1 in NK cells is a key immunometabolic feature significantly associated with the risk of nosocomial infection in patients with CCI, and suggest that NK cell-specific immunometabolic features may be applied for assessing the risk of nosocomial infection development.

**Figure 5:**
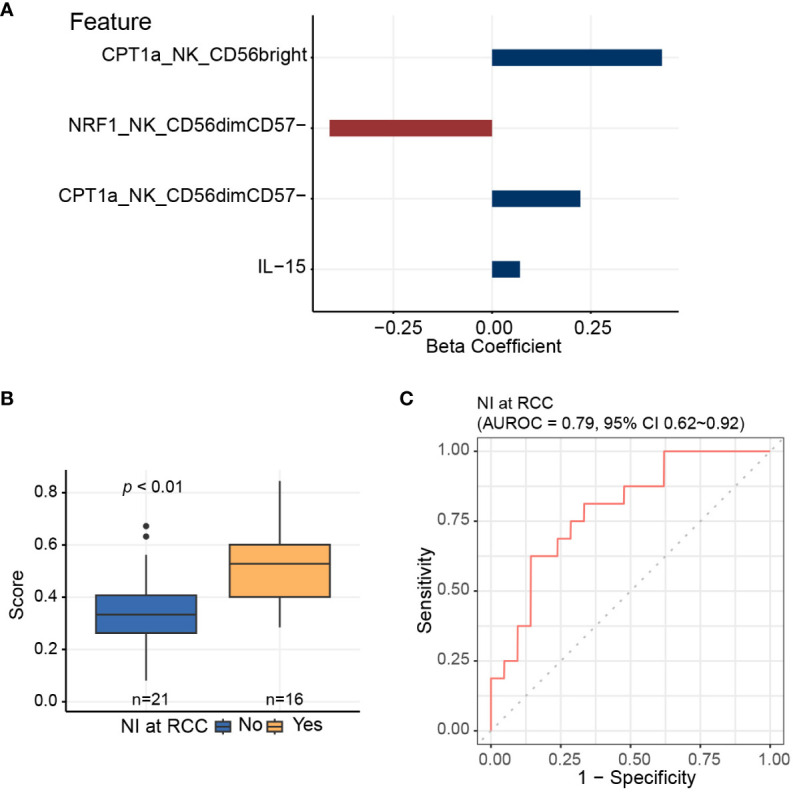
Elastic net logistic regression is applied to identify NK cell-specific features predictive of nosocomial infection risk. **(A)** Three of 11 NK cell-related features, which are significantly associated with coordinate 3 of the multidimensional scaling plot, are selected using elastic net algorithm, and are included to generate the predictive score for assessing the risk of nosocomial infection. **(B)** Predictive scores based on elastic net models for patients who develop nosocomial infection and those who do not. Horizontal line indicates median, boxes indicate interquartile range, and the upper and lower whiskers extended to the largest and the smallest value at most 1.5 interquartile range from the upper and the lower hinges, respectively. Outliers beyond the ends of the whiskers are plotted individually. The *p* value is calculated by the Mann-Whitney U test. **(C)** Area under receiver operating characteristics curve (AUROC) is calculated for assessing the performance of the predictive score in evaluating the risk of nosocomial infection.

Since the data is inherently high dimensional with the number of features much greater than the number of patients, we performed elastic net logistic regression to identify NK cell-specific immunometabolic features that have potential as biomarkers to predict the risk of nosocomial infection. This analysis indicated that CPT1a and NRF1 expression levels in NK cell subsets and plasma IL-15 levels are strongly associated with nosocomial infection risk (**Figures 5A, B**). The AUROC for distinguishing those subjects likely to develop nosocomial infection using these three features was 0.79 (95% CI of 0.62-0.92, **Figure 5C**).

### Mitochondrial fatty acid oxidation and biogenesis in NK cells are associated with the severity and clinical outcomes of critical COVID-19 infection

**Figure 6:**
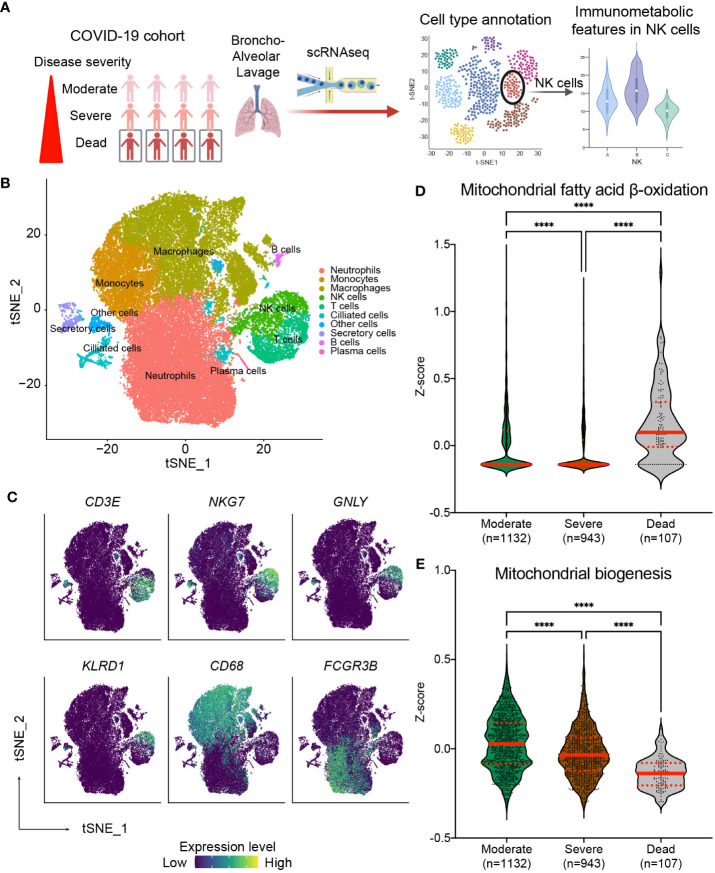
Correlation of NK cell-specific immunometabolic features with the disease severity and clinical outcome in critical COVID-19 infection. **(A)** Diagram outlining the method for validation utilizing publicly available single-cell RNA sequencing (scRNA-seq) data from fourteen COVID-19 patients including three from the moderate group, six from the severe group, and five from the deceased group. **(B)** t-distributed stochastic neighbor embedding (tSNE) plots of scRNA-seq data showing the major cell types, each labeled with a distinct color. **(C)** The lineage specific marker genes for each cell types are shown. The expression levels of indicated genes are color coded. **(D, E)** Relative expression of the mitochondrial fatty acid β-oxidation pathway **(D)** and the mitochondrial biogenesis pathway **(E)** across patient groups are exhibited through violin plots of z-scores for genes involved in specific pathways. The lines indicate median and interquartile range. The *p* values are calculated using Kruskal-Wallis test and adjusted for multiple comparisons using the Dunn’s method. (**** *p*< 0.0001).

Our results above suggest that the risk of nosocomial infection in CCI is significantly correlated with the expression of CPT1a and NRF1 in NK cells, and the findings suggest that NK cell-specific mitochondrial fatty acid oxidation and biogenesis might be crucial in host immunity against invading pathogens (37–39). Several recent studies reported increased risk of secondary infection in critical ill patients with COVID-19 infection, and the occurrence of secondary infection remarkably raises the mortality rate of COVID-19-infected critically ill patients (40–45). On the basis of our findings, we surmised that the severity and outcome of critical COVID-19 infection may be correlated with NK cell-specific metabolic features, in particular mitochondrial biogenesis and fatty acid oxidation. To further investigate our assumption, we integrated and clustered scRNA-seq data from two publicly available datasets to assess whether the immunometabolic patterns in NK cells in bronchoalveolar lavage fluid correlate with disease severity and the survival in COVID-19 patients (**Figure 6A**) (33, 34). We identified seven transcriptional clusters (**Figures 6B, C**). Among these clusters, the NK cell population was distinguished by the enrichment of NK cell-specific markers including *NKG7* and *GNLY*. To investigate the involvement of the NRF1-associated mitochondria biogenesis pathway and the CPT1a-related fatty acid oxidation pathway in NK cells, we employed established gene sets for these pathways to calculate the expression levels of genes in these pathways in each single cell. Notably, the fatty acid oxidation signature scores were highest in NK cells of patients who succumbed to infection (**Figure 6D**). Conversely, the mitochondria biogenesis scores were significantly higher in NK cells of patients with moderate or severe COVID-19 infection compared to those who did not survive infection (**Figure 6E**). To affirm that our observed findings are not a consequence of batch effects or sampling bias, we conducted a validation analysis on another independent cohort (36) (**Supplementary Figures 6A, B**). Similarly, the results showed the signature of upregulated mitochondrial fatty acid oxidation and downregulated mitochondrial biogenesis in circulating NK cells is associated with mortality of critical COVID-19 infection (**Supplementary Figures 6C, D**). The results from the above scRNA-seq analyses together reveal that immunometabolic features, particularly mitochondrial fatty acid oxidation and biogenesis, in NK cells are correlated with disease severity and clinical outcomes in critical COVID-19 illness. Furthermore, the findings from our cohort and the analyses of scRNA-seq datasets suggest the immunometabolic regulation in NK cells may be a crucial aspect of host immunity in critical infectious illness.

## Discussion

The role of NK cells in host defense against microbial infection during critical illness remains unclear. Here, through unbiased exploration, we uncovered the association between NK cell-specific immunometabolism, in particular the altered expression of NRF1 and CPT1a, and nosocomial infection in patients with CCI. The findings are strongly supported by scRNA-seq analysis results in COVID-19-infected population, and bring forward the tenet about NK cell-specific immunometabolic dysregulation in host immunity against microbial invasion in critical illness. Characterizing NK cell-specific immunometabolic features may be applied for assessing the risk of nosocomial infection to select susceptible critically ill subjects for infection preventive interventions.

Several previous studies have explored features of immunosuppression in critically ill patients, and have demonstrated a reduction in T lymphocytes, along with an increased expression of inhibitory receptors, such as PD-1 and CTLA-4 in T lymphocytes and the presence of ligands like PD-L1 in monocytes and dendritic cells. Furthermore, patients post sepsis or septic shock often exhibit diminished monocytic expression of HLA-DR and other immunosuppressive features (46–48). The association between these immunosuppressive features and the risk of nosocomial infection is not fully explored, although some studies revealed that monocytic expression of PD-1 and HLA-DR may be associated with the risk of nosocomial infection after sepsis (21, 49). However, in our study, the abundance and the surface marker expression of immune cells are not correlated with the risk of nosocomial infection. We attribute the disparities in findings between studies to the inherent heterogeneity within the critically ill population, coupled with the dynamic nature of these patients and the challenge posed by small sample sizes. Additionally, most of previous studies mainly focused on patients with sepsis. Through extensive mass cytometric profiling, we found that NK cell-specific immunometabolic alterations were significantly associated with the risk of nosocomial infection. Two subsets of NK cells, those that express high levels of CD56 and those that express low levels of CD56, exist in the human blood; the latter subset constitutes 90% of the NK cell population (50). NK cells that express low levels of CD56 are mainly cytotoxic, whereas NK cells that express high levels of CD56 produce cytokines, such as interferon (IFN) γ and IL-10, after activation (51). In a murine model, clearance of secondary *Pseudomonas* infection is hampered after sepsis induction by cecal ligation and puncture due to impaired IFNγ production from NK cells (52). In addition, a recent study revealed that impaired IFNγ production of NK cells in is associated with nosocomial infection in critically ill patients following a systemic inflammatory response (22). Therefore, our findings, together with the supporting evidence from previous studies, suggest the critical role of NK cell-mediated immunity in protection against invading pathogens and nosocomial infection in critically ill patients and further indicate that predictive scores characterizing altered NK cell immunity may enable stratification for risk of nosocomial infection in critically ill population.

The proper function of NK cells relies on metabolic control (53–55). Glycolysis and oxidative phosphorylation are required to support NK cell function after activation (53, 54). A previous study demonstrated that PGC1α loss in NK cells would suppress mitochondrial oxidative phosphorylation, leading to compromised cytotoxic potential and cytokine production (37). Although our data revealed that PGC1α expression in NK cells is not associated with the risk of nosocomial infection in CCI, the activity of NRF1 is regulated by PGC1α, and NRF1 regulates the nuclear genomic transcription of genes related to respiratory complexes, mitochondrial protein transport, mitochondrial genomic transcription, and protein translation (38). Decreased NRF1 expression in NK cells may thus impair mitochondrial bioenergetics and alter the effector function of NK cells. In addition, depleting NRF1 has been shown to upregulate lipid metabolism (39), and, in line with this regulatory axis, our data implicate CPT1a, which regulates mitochondrial import and β oxidation of long-chain fatty acids, as an immunometabolic regulator associated with the risk of nosocomial infection in critical illness. Although our data also revealed elevated CPT1a expression not only in NK cells but also in NKT cells and some T lymphocyte subsets among patients with nosocomial infection, it remains unclear whether CPT1a is essential for T lymphocyte function, based on the results from transgenic murine model with T lymphocyte-specific deletion of CPT1a (56). Likewise, the impact of mitochondrial fatty acid oxidation on the effector function of NK cells is heavily contingent on context. Several studies have indicated that the augmentation of NK cell activation and effector function through IL-10 or IL-15 stimulation is reliant on the upregulation of mitochondrial fatty acid oxidation (57, 58). Conversely, another study demonstrated that increased lipid metabolism and lipid transport into the mitochondria result in NK cell dysfunction (55). A recent study by Liu C et al. applied scRNA-seq to explore the transcriptomic features of circulating immune cells in critical COVID-19 infection, and uncovered that mortality is associated with a metabolic signature of increased fatty acid metabolism in NK cells (36). Our data and the findings from the study by Liu C et al. (36) together suggest that lipid metabolism in NK cells may be a prognostic biomarker in critical infectious illness. However, studies are required to fully resolve whether the metabolic reprogramming associated with NK cell activation is essential to NK cell immunity, and to elucidate the roles of NK cell-specific NRF1 and CPT1a in host immunity against infection.

In the context of an immune response, immune cells dynamically interact with their milieu, utilizing surface receptors to interpret extracellular prompts and recalibrate intracellular homeostasis to effectively neutralize pathogenic threats. One critical facet of this internal recalibration is the adaptation of cellular metabolism, tailored to meet the immediate energy requisites and strategic objectives of the immune response (59). For example, in the acute phase, T cells undergo rapid proliferation and synthesize pivotal effector molecules, necessitating a surge in bioenergetic and biosynthetic pathways (60). Conversely, upon pathogen clearance, these activated immune components transition, necessitating metabolic reprogramming to support roles such as memory cell formation or tissue repair functions (61). This metabolic versatility encompasses a shift from glycolysis during the acute phase—an energy-lavish pathway suitable for immediate cellular demands—to a more energy-conservative fatty acid oxidation process post-threat, aligning with the cells’ long-term functional commitments.

Similarly, the activation mechanics of NK cells are governed by a sophisticated network of checks and balances involving both activating and inhibitory receptors, nuanced by additional layers of control from cytokines and available nutrients (62–64). Intriguingly, metabolic reprogramming in NK cells is not uniform but stimulus-specific (65, 66). For instance, IFNγ production, when induced through activation receptors, mandates a glucose-intensive oxidative phosphorylation pathway, contrasting with cytokine-stimulated IFNγ production (e.g., in the presence of IL-12 and IL-18) that proceeds independently of glycolytic pathways (67). Our research underscores the clinical ramifications of these metabolic nuances, linking basal metabolic profiles of NK cells with clinical outcomes such as the prevalence of nosocomial infection in patients with critical care illnesses and the efficacy of infection management in SARS-Co-V2 cases. These insights accentuate the need for a holistic exploration of NK cell metabolic landscapes to fully comprehend their immunological comportments.

Extending beyond metabolic perspectives, the NK cell receptor repertoire—crucial for defining the host’s immunological countermeasures—is demonstrably sculpted by a spectrum of viral pathogens, including but not limited to HIV, human cytomegalovirus, and SARS-Co-V2 (68–81). Concurrently, it has been reported that a troubling association between chronic viral infection, particularly HIV, and the perturbation of NK cell mitochondrial integrity, manifesting as compromised oxidative phosphorylation, escalated mitochondrial depolarization, and conspicuous mitochondrial fragmentation (82). These phenomena suggest a dual impact of viral pathogens on NK cells, implicating concurrent modulations in receptor architecture and metabolic comportment. Therefore, prospective research endeavors dissecting the interdependencies between metabolic processes and receptor configurations in NK cells hold promise for innovative immunological interventions aimed at optimizing viral control mechanisms.

The mechanisms leading to NK cell dysfunction after systemic inflammation are not fully understood and may be related to both intrinsic defects, such as decreased expression of IL-12 receptor, and extrinsic regulation, such as growth and differentiation factor GDF-15 (22). Meanwhile, while activation of NK cells is associated with upregulated mitochondrial fatty acid oxidation and oxidative phosphorylation, continuous stimulation is found to hamper mitochondrial bioenergetics and causes NK cell exhaust (58, 65). In this study, the data suggest that occurrence of nosocomial infection in CCI is associated with increased IL-10 and IL-15 levels in the circulation. The altered cytokine profile and upregulated CPT1a expression in NK cells may thus suggest prolonged activation of NK cells, resulting in impaired NK cell immunity (58). Besides, evidence suggests that myeloid-derived suppressor cells (MDSCs) may be crucial for immune dysfunction and nosocomial infection in CCI (83). A persistent increase in circulatory MDSCs is found after sepsis and septic shock and is significantly associated with an increased risk of nosocomial infection in CCI (16). MDSCs, including granulocytic and monocytic subsets, can suppress T cell function through arginine deprivation in the microenvironment by upregulation of arginase 1 and nitric oxide synthase, and the production of nitrogen oxide, reactive oxygen species, and peroxynitrite (84). Furthermore, MDSCs were found to suppress the development, cytotoxicity, and IFNγ production of NK cells in murine models (85, 86). Although further studies will be required to confirm the immunoregulatory interactions between MDSCs and NK cells in human subjects, the murine studies suggest a potential causal link between the emergence of MDSCs in patients with CCI and altered immunometabolism in NK cells. The detailed trajectory of immunometabolic alterations in NK cells after septic shock and the exact mechanisms that results in metabolic rewiring of NK cells in the CCI population are unclear, and warrant future studies to clarify.

This study has some limitations. First, this is a prospective study with an exploratory aim to identify immunometabolic features associated with nosocomial infection in CCI. Although our analyses using mass cytometry data were comprehensive and were confirmed in an independent cohort, the sample size of our study population is small. In addition, immunometabolic characterization at different time points will further provide valuable trajectory insights into the immunometabolic signature associated with nosocomial infection in critical illness. Further studies are needed to validate our findings, and to uncover the dynamics of immunometabolism in critical illness. Second, for exploratory purposes, we assessed the metabolic regulators of various immune cells in blood samples. The association between the risk of nosocomial infection in CCI and several NK cell-specific features other than metabolic changes, such as surface marker expression, regulatory cytokine release, and effector functions, needs further investigation. Incorporation of additional features may result in more accurate identification of critically ill patients at a high risk of nosocomial infection. Third, the immunity of patients with CCI is probably influenced by both the underlying comorbidities and clinical course of the critical illness. Furthermore, dysfunction of NK cells can be observed in patients with major trauma (22), and the findings suggest that changes in the NRF1 and CPT1a regulatory axis may occur in patients with CCI patients following non-septic systemic inflammation. Thus, the clinical features predisposing to NK cell-specific immunometabolic changes require further research.

In conclusion, our findings shed light on the role of perturbed NK cell immunometabolism, including NRF1 downregulation and CPT1a upregulation, in the risk of nosocomial infection in CCI. Further studies are required to characterize NK cell-specific immunity in critically ill patients and to explore the risk factors leading to NK cell dysfunction in CCI.

## Data availability statement

The original contributions presented in the study are included in the article/**Supplementary Material**, further inquiries can be directed to the corresponding authors.

## Ethics statement

The studies involving humans were approved by the Institutional Review Board of National Taiwan University Hospital. The studies were conducted in accordance with the local legislation and institutional requirements. The participants provided their written informed consent to participate in this study.

## Author contributions

K-PC: Conceptualization, Data curation, Formal analysis, Funding acquisition, Investigation, Methodology, Visualization, Writing – original draft, Writing – review & editing. J-YS: Formal analysis, Writing – original draft, Writing – review & editing. Y-FW: Data curation, Formal analysis, Methodology, Visualization, Writing – original draft, Writing – review & editing. BB: Formal analysis, Writing – original draft, Writing – review & editing. Y-CY: Investigation, Resources, Visualization, Writing – review & editing. JC-C: Data curation, Investigation, Project administration, Resources, Writing – review & editing. L-TK: Data curation, Investigation, Resources, Writing – review & editing. Y-JC: Investigation, Project administration, Writing – review & editing. Y-TL: Investigation, Methodology, Writing – review & editing. Y-HJ: Investigation, Project administration, Writing – review & editing. KN: Investigation, Visualization, Writing – review & editing. S-YR: Investigation, Visualization, Writing – review & editing. J-YC: Investigation, Visualization, Writing – review & editing. H-TC: Investigation, Visualization, Writing – review & editing. J-SJ: Investigation, Visualization, Writing – review & editing. Y-TH: Formal analysis, Investigation, Methodology, Visualization, Writing – original draft, Writing – review & editing. S-YC: Conceptualization, Data curation, Formal analysis, Funding acquisition, Investigation, Methodology, Resources, Software, Supervision, Visualization, Writing – original draft, Writing – review & editing. C-JY: Conceptualization, Funding acquisition, Investigation, Resources, Supervision, Visualization, Writing – review & editing.

## References

[1] Nelson JE Cox CE Hope AA Carson SS. Chronic critical illness. Am J Respir Crit Care Med (2010) 182(4):446–54. doi: 10.1164/rccm.201002-0210CI PMC2937238 20448093

[2] Iwashyna TJ Hodgson CL Pilcher D Bailey M van Lint A Chavan S. Timing of onset and burden of persistent critical illness in Australia and New Zealand: a retrospective, population-based, observational study. Lancet Respir Med (2016) 4(7):566–73. doi: 10.1016/S2213-2600(16)30098-4 27155770

[3] Kahn JM Le T Angus DC Cox CE Hough CL White DB. The epidemiology of chronic critical illness in the United States*. Crit Care Med (2015) 43(2):282–7. doi: 10.1097/CCM.0000000000000710 PMC7901538 25377018

[4] Scheinhorn DJ Hassenpflug MS Votto JJ Chao DC Epstein SK Doig GS. Post-ICU mechanical ventilation at 23 long-term care hospitals: a multicenter outcomes study. Chest (2007) 131(1):85–93. doi: 10.1378/chest.06-1081 17218560

[5] Hu D Ren J Wang G Gu G Chen J Zhou B. Persistent inflammation-immunosuppression catabolism syndrome, a common manifestation of patients with enterocutaneous fistula in intensive care unit. J Trauma Acute Care Surg (2014) 76(3):725–9. doi: 10.1097/TA.0b013e3182aafe6b 24553541

[6] Zilberberg MD Nathanson BH Puzniak LA Shorr AF. Descriptive epidemiology and outcomes of nonventilated hospital-acquired, ventilated hospital-acquired, and ventilator-associated bacterial pneumonia in the United States, 2012-2019. Crit Care Med (2022) 50(3):460–8. doi: 10.1097/CCM.0000000000005298 PMC8855942 34534129

[7] Zhu S Kang Y Wang W Cai L Sun X Zong Z. The clinical impacts and risk factors for non-central line-associated bloodstream infection in 5046 intensive care unit patients: an observational study based on electronic medical records. Crit Care (2019) 23(1):52. doi: 10.1186/s13054-019-2353-5 30777109 PMC6379966

[8] Marchioni A Tonelli R Sdanganelli A Gozzi F Musaro L Fantini R. Prevalence and development of chronic critical illness in acute patients admitted to a respiratory intensive care setting. Pulmonology (2020) 26(3):151–8. doi: 10.1016/j.pulmoe.2019.09.006 31672594

[9] Cox MC Brakenridge SC Stortz JA Hawkins RB Darden DB Ghita GL. Abdominal sepsis patients have a high incidence of chronic critical illness with dismal long-term outcomes. Am J Surg (2020) 220(6):1467–74. doi: 10.1016/j.amjsurg.2020.07.016 PMC7731616 32807383

[10] Yende S D’Angelo G Kellum JA Weissfeld L Fine J Welch RD. Inflammatory markers at hospital discharge predict subsequent mortality after pneumonia and sepsis. Am J Respir Crit Care Med (2008) 177(11):1242–7. doi: 10.1164/rccm.200712-1777OC PMC2720087 18369199

[11] Yende S Kellum JA Talisa VB Peck Palmer OM Chang CH Filbin MR. Long-term host immune response trajectories among hospitalized patients with sepsis. JAMA Netw Open (2019) 2(8):e198686. doi: 10.1001/jamanetworkopen.2019.8686 31390038 PMC6686981

[12] Gentile LF Cuenca AG Efron PA Ang D Bihorac A McKinley BA. Persistent inflammation and immunosuppression: a common syndrome and new horizon for surgical intensive care. J Trauma Acute Care Surg (2012) 72(6):1491–501. doi: 10.1097/TA.0b013e318256e000 PMC3705923 22695412

[13] Mankowski RT Anton SD Ghita GL Brumback B Darden DB Bihorac A. Older adults demonstrate biomarker evidence of the persistent inflammation, immunosuppression and catabolism syndrome (PICS) after sepsis. J Gerontol A Biol Sci Med Sci (2021) 77(1):188–96. doi: 10.1093/gerona/glab080 PMC8751807 33721883

[14] Darden DB Bacher R Brusko MA Knight P Hawkins RB Cox MC. Single-cell RNA-seq of human myeloid-derived suppressor cells in late sepsis reveals multiple subsets with unique transcriptional responses: A pilot study. Shock (2021) 55(5):587–95. doi: 10.1097/SHK.0000000000001671 PMC8019679 33021571

[15] Hollen MK Stortz JA Darden D Dirain ML Nacionales DC Hawkins RB. Myeloid-derived suppressor cell function and epigenetic expression evolves over time after surgical sepsis. Crit Care (2019) 23(1):355. doi: 10.1186/s13054-019-2628-x 31722736 PMC6854728

[16] Mathias B Delmas AL Ozrazgat-Baslanti T Vanzant EL Szpila BE Mohr AM. Human myeloid-derived suppressor cells are associated with chronic immune suppression after severe sepsis/septic shock. Ann Surg (2017) 265(4):827–34. doi: 10.1097/SLA.0000000000001783 PMC5102824 27163951

[17] Bjorkstrom NK Strunz B Ljunggren HG. Natural killer cells in antiviral immunity. Nat Rev Immunol (2022) 22(2):112–23. doi: 10.1038/s41577-021-00558-3 PMC8194386 34117484

[18] Li X Wenes M Romero P Huang SC-C Fendt S-M Ho P-C. Navigating metabolic pathways to enhance antitumour immunity and immunotherapy. Nat Rev Clin Oncol (2019) 16(7):425–41. doi: 10.1038/s41571-019-0203-7 30914826

[19] Cheng SC Scicluna BP Arts RJ Gresnigt MS Lachmandas E Giamarellos-Bourboulis EJ. Broad defects in the energy metabolism of leukocytes underlie immunoparalysis in sepsis. Nat Immunol (2016) 17(4):406–13. doi: 10.1038/ni.3398 26950237

[20] Uhel F Azzaoui I Gregoire M Pangault C Dulong J Tadie JM. Early expansion of circulating granulocytic myeloid-derived suppressor cells predicts development of nosocomial infections in patients with sepsis. Am J Respir Crit Care Med (2017) 196(3):315–27. doi: 10.1164/rccm.201606-1143OC 28146645

[21] Guignant C Lepape A Huang X Kherouf H Denis L Poitevin F. Programmed death-1 levels correlate with increased mortality, nosocomial infection and immune dysfunctions in septic shock patients. Crit Care (2011) 15(2):R99. doi: 10.1186/cc10112 21418617 PMC3219369

[22] Kleinertz H Hepner-Schefczyk M Ehnert S Claus M Halbgebauer R Boller L. Circulating growth/differentiation factor 15 is associated with human CD56(bright) natural killer cell dysfunction and nosocomial infection in severe systemic inflammation. EBioMedicine (2019) 43:380–91. doi: 10.1016/j.ebiom.2019.04.018 PMC6557805 30992245

[23] Hartmann FJ Mrdjen D McCaffrey E Glass DR Greenwald NF Bharadwaj A. Single-cell metabolic profiling of human cytotoxic T cells. Nat Biotechnol (2021) 39(2):186–97. doi: 10.1038/s41587-020-0651-8 PMC7878201 32868913

[24] Voss K Hong HS Bader JE Sugiura A Lyssiotis CA Rathmell JC. A guide to interrogating immunometabolism. Nat Rev Immunol (2021) 21(10):637–52. doi: 10.1038/s41577-021-00529-8 PMC8478710 33859379

[25] Levine LS Hiam-Galvez KJ Marquez DM Tenvooren I Madden MZ Contreras DC. Single-cell analysis by mass cytometry reveals metabolic states of early-activated CD8+ T cells during the primary immune response. Immunity (2021) 54(4):829–844.e5. doi: 10.1016/j.immuni.2021.02.018 33705706 PMC8046726

[26] Keng LT Chung KP Lin SY Liang SK Cheng JC Chen IC. Significant clinical factors associated with long-term mortality in critical cancer patients requiring prolonged mechanical ventilation. Sci Rep (2017) 7(1):2148. doi: 10.1038/s41598-017-02418-4 28526862 PMC5438375

[27] Chung KP Chen GY Chuang TY Huang YT Chang HT Chen YF. Increased plasma acetylcarnitine in sepsis is associated with multiple organ dysfunction and mortality: A multicenter cohort study. Crit Care Med (2019) 47(2):210–8. doi: 10.1097/CCM.0000000000003517 30379669

[28] Force ADT Ranieri VM Rubenfeld GD Thompson BT Ferguson ND Caldwell E. Acute respiratory distress syndrome: the Berlin Definition. JAMA (2012) 307(23):2526–33. doi: 10.1001/jama.2012.5669 22797452

[29] Kruskal JB Wish M. Multidimensional scaling. Sage university paper series on quantitative applications in the social sciences, 07-011. Thousand Oaks, CA, USA: SAGE Publications, INC. (1978). doi: 10.4135/9781412985130.

[30] Saito T Rehmsmeier M. Precrec: fast and accurate precision-recall and ROC curve calculations in R. Bioinformatics (2017) 33(1):145–7. doi: 10.1093/bioinformatics/btw570 PMC5408773 27591081

[31] Storey JD Tibshirani R. Statistical significance for genomewide studies. Proc Natl Acad Sci USA (2003) 100(16):9440–5. doi: 10.1073/pnas.1530509100 PMC170937 12883005

[32] Friedman J Hastie T Tibshirani R. Regularization paths for generalized linear models via coordinate descent. J Stat Softw (2010) 33(1):1–22. doi: 10.18637/jss.v033.i01 20808728 PMC2929880

[33] Liao M Liu Y Yuan J Wen Y Xu G Zhao J. Single-cell landscape of bronchoalveolar immune cells in patients with COVID-19. Nat Med (2020) 26(6):842–4. doi: 10.1038/s41591-020-0901-9 32398875

[34] Bost P De Sanctis F Cane S Ugel S Donadello K Castellucci M. Deciphering the state of immune silence in fatal COVID-19 patients. Nat Commun (2021) 12(1):1428. doi: 10.1038/s41467-021-21702-6 33674591 PMC7935849

[35] Hasan MZ Islam S Matsumoto K Kawai T. Meta-analysis of single-cell RNA-seq data reveals phenotypic switching of immune cells in severe COVID-19 patients. Comput Biol Med (2021) 137:104792. doi: 10.1016/j.compbiomed.2021.104792 34478921 PMC8390121

[36] Liu C Martins AJ Lau WW Rachmaninoff N Chen J Imberti L. Time-resolved systems immunology reveals a late juncture linked to fatal COVID-19. Cell (2021) 184(7):1836–1857 e22. doi: 10.1016/j.cell.2021.02.018 33713619 PMC7874909

[37] Gerbec ZJ Hashemi E Nanbakhsh A Holzhauer S Yang C Mei A. Conditional deletion of PGC-1alpha results in energetic and functional defects in NK cells. iScience (2020) 23(9):101454. doi: 10.1016/j.isci.2020.101454 32858341 PMC7474003

[38] Scarpulla RC. Transcriptional paradigms in mammalian mitochondrial biogenesis and function. Physiol Rev (2008) 88(2):611–38. doi: 10.1152/physrev.00025.2007 18391175

[39] Tsujita T Peirce V Baird L Matsuyama Y Takaku M Walsh SV. Transcription factor Nrf1 negatively regulates the cystine/glutamate transporter and lipid-metabolizing enzymes. Mol Cell Biol (2014) 34(20):3800–16. doi: 10.1128/MCB.00110-14 PMC4187719 25092871

[40] De Bruyn A Verellen S Bruckers L Geebelen L Callebaut I De Pauw I. Secondary infection in COVID-19 critically ill patients: a retrospective single-center evaluation. BMC Infect Dis (2022) 22(1):207. doi: 10.1186/s12879-022-07192-x 35236299 PMC8890021

[41] Adelman MW Bhamidipati DR Hernandez-Romieu AC Babiker A Woodworth MH Robichaux C. Clinical research collaborative: secondary bacterial pneumonias and bloodstream infections in patients hospitalized with COVID-19. Ann Am Thorac Soc (2021) 18(9):1584–7. doi: 10.1513/AnnalsATS.202009-1093RL PMC8489870 33823119

[42] Na YS Baek AR Baek MS Kim WY Kim JH Lee BY. Clinical outcomes of and risk factors for secondary infection in patients with severe COVID-19: a multicenter cohort study in South Korea. Korean J Intern Med (2023) 38(1):68–79. doi: 10.3904/kjim.2022.084 36420564 PMC9816674

[43] Shafran N Shafran I Ben-Zvi H Sofer S Sheena L Krause I. Secondary bacterial infection in COVID-19 patients is a stronger predictor for death compared to influenza patients. Sci Rep (2021) 11(1):12703. doi: 10.1038/s41598-021-92220-0 34135459 PMC8209102

[44] Pourajam S Kalantari E Talebzadeh H Mellali H Sami R Soltaninejad F. Secondary bacterial infection and clinical characteristics in patients with COVID-19 admitted to two intensive care units of an academic hospital in Iran during the first wave of the pandemic. Front Cell Infect Microbiol (2022) 12:784130. doi: 10.3389/fcimb.2022.784130 35281440 PMC8904895

[45] Ripa M Galli L Poli A Oltolini C Spagnuolo V Mastrangelo A. Secondary infections in patients hospitalized with COVID-19: incidence and predictive factors. Clin Microbiol Infect (2021) 27(3):451–7. doi: 10.1016/j.cmi.2020.10.021 PMC7584496 33223114

[46] Gogos C Kotsaki A Pelekanou A Giannikopoulos G Vaki I Maravitsa P. Early alterations of the innate and adaptive immune statuses in sepsis according to the type of underlying infection. Crit Care (2010) 14(3):R96. doi: 10.1186/cc9031 20504311 PMC2911733

[47] Boomer JS Shuherk-Shaffer J Hotchkiss RS Green JM. A prospective analysis of lymphocyte phenotype and function over the course of acute sepsis. Crit Care (2012) 16(3):R112. doi: 10.1186/cc11404 22742734 PMC3580670

[48] Boomer JS To K Chang KC Takasu O Osborne DF Walton AH. Immunosuppression in patients who die of sepsis and multiple organ failure. JAMA (2011) 306(23):2594–605. doi: 10.1001/jama.2011.1829 PMC3361243 22187279

[49] Leijte GP Rimmele T Kox M Bruse N Monard C Gossez M. Monocytic HLA-DR expression kinetics in septic shock patients with different pathogens, sites of infection and adverse outcomes. Crit Care (2020) 24(1):110. doi: 10.1186/s13054-020-2830-x 32192532 PMC7082984

[50] Freud AG Mundy-Bosse BL Yu J Caligiuri MA. The broad spectrum of human natural killer cell diversity. Immunity (2017) 47(5):820–33. doi: 10.1016/j.immuni.2017.10.008 PMC5728700 29166586

[51] Cooper MA Fehniger TA Caligiuri MA. The biology of human natural killer-cell subsets. Trends Immunol (2001) 22(11):633–40. doi: 10.1016/s1471-4906(01)02060-9 11698225

[52] Pastille E Pohlmann S Wirsdorfer F Reib A Flohe SB. A disturbed interaction with accessory cells upon opportunistic infection with Pseudomonas aeruginosa contributes to an impaired IFN-gamma production of NK cells in the lung during sepsis-induced immunosuppression. Innate Immun (2015) 21(2):115–26. doi: 10.1177/1753425913517274 24406749

[53] Keppel MP Saucier N Mah AY Vogel TP Cooper MA. Activation-specific metabolic requirements for NK Cell IFN-gamma production. J Immunol (2015) 194(4):1954–62. doi: 10.4049/jimmunol.1402099 PMC4323953 25595780

[54] Cong J Wang X Zheng X Wang D Fu B Sun R. Dysfunction of natural killer cells by FBP1-induced inhibition of glycolysis during lung cancer progression. Cell Metab (2018) 28(2):243–255 e5. doi: 10.1016/j.cmet.2018.06.021 30033198

[55] Michelet X Dyck L Hogan A Loftus RM Duquette D Wei K. Metabolic reprogramming of natural killer cells in obesity limits antitumor responses. Nat Immunol (2018) 19(12):1330–40. doi: 10.1038/s41590-018-0251-7 30420624

[56] Raud B Roy DG Divakaruni AS Tarasenko TN Franke R Ma EH. Etomoxir actions on regulatory and memory T cells are independent of cpt1a-mediated fatty acid oxidation. Cell Metab (2018) 28(3):504–15.e7. doi: 10.1016/j.cmet.2018.06.002 30043753 PMC6747686

[57] Wang Z Guan D Huo J Biswas SK Huang Y Yang Y. IL-10 Enhances Human Natural Killer Cell Effector Functions via Metabolic Reprogramming Regulated by mTORC1 Signaling. Front Immunol (2021) 12:619195. doi: 10.3389/fimmu.2021.619195 33708210 PMC7940510

[58] Felices M Lenvik AJ McElmurry R Chu S Hinderlie P Bendzick L. Continuous treatment with IL-15 exhausts human NK cells via a metabolic defect. JCI Insight (2018) 3(3):e96219. doi: 10.1172/jci.insight.96219 29415897 PMC5821201

[59] O’Neill LA Kishton RJ Rathmell J. A guide to immunometabolism for immunologists. Nat Rev Immunol (2016) 16(9):553–65. doi: 10.1038/nri.2016.70 PMC5001910 27396447

[60] Makowski L Chaib M Rathmell JC. Immunometabolism: From basic mechanisms to translation. Immunol Rev (2020) 295(1):5–14. doi: 10.1111/imr.12858 32320073 PMC8056251

[61] Wik JA Skalhegg BS. T cell metabolism in infection. Front Immunol (2022) 13:840610. doi: 10.3389/fimmu.2022.840610 35359994 PMC8964062

[62] Long EO Kim HS Liu D Peterson ME Rajagopalan S. Controlling natural killer cell responses: integration of signals for activation and inhibition. Annu Rev Immunol (2013) 31:227–58. doi: 10.1146/annurev-immunol-020711-075005 PMC3868343 23516982

[63] Smyth MJ Cretney E Kelly JM Westwood JA Street SE Yagita H. Activation of NK cell cytotoxicity. Mol Immunol (2005) 42(4):501–10. doi: 10.1016/j.molimm.2004.07.034 15607806

[64] Martinet L Smyth MJ. Balancing natural killer cell activation through paired receptors. Nat Rev Immunol (2015) 15(4):243–54. doi: 10.1038/nri3799 25743219

[65] Osuna-Espinoza KY Rosas-Taraco AG. Metabolism of NK cells during viral infections. Front Immunol (2023) 14:1064101. doi: 10.3389/fimmu.2023.1064101 36742317 PMC9889541

[66] O’Brien KL Finlay DK. Immunometabolism and natural killer cell responses. Nat Rev Immunol (2019) 19(5):282–90. doi: 10.1038/s41577-019-0139-2 30808985

[67] Keppel MP Saucier N Mah AY Vogel TP Cooper MA. Activation-specific metabolic requirements for NK cell IFN-γ Production. J Immunol (2015) 194(4):1954–62. doi: 10.4049/jimmunol.1402099 PMC4323953 25595780

[68] Béziat V Liu LL Malmberg J-A Ivarsson MA Sohlberg E Björklund AT. NK cell responses to cytomegalovirus infection lead to stable imprints in the human KIR repertoire and involve activating KIRs. Blood (2013) 121(14):2678–88. doi: 10.1182/blood-2012-10-459545 PMC3617633 23325834

[69] Béziat V Dalgard O Asselah T Halfon P Bedossa P Boudifa A. CMV drives clonal expansion of NKG2C+ NK cells expressing self-specific KIRs in chronic hepatitis patients. Eur J Immunol (2012) 42(2):447–57. doi: 10.1002/eji.201141826 22105371

[70] Saghafian-Hedengren S Sohlberg E Theorell J Carvalho-Queiroz C Nagy N Persson J-O. Epstein-barr virus coinfection in children boosts cytomegalovirus-induced differentiation of natural killer cells. J Virol (2013) 87(24):13446–55. doi: 10.1128/jvi.02382-13 PMC3838261 24089567

[71] Strauss-Albee DM Fukuyama J Liang EC Yao Y Jarrell JA Drake AL. Human NK cell repertoire diversity reflects immune experience and correlates with viral susceptibility. Sci Trans Med (2015) 7(297):297ra115. doi: 10.1126/scitranslmed.aac5722 PMC4547537 26203083

[72] Hsieh WC Lai EY Liu YT Wang YF Tzeng YS Cui L. NK cell receptor and ligand composition influences the clearance of SARS-CoV-2. J Clin Invest (2021) 131(21):e146408. doi: 10.1172/JCI146408 34720095 PMC8553551

[73] Petitdemange C Becquart P Wauquier N Béziat V Debré P Leroy EM. Unconventional Repertoire Profile Is Imprinted during Acute Chikungunya Infection for Natural Killer Cells Polarization toward Cytotoxicity. PloS Pathog (2011) 7(9):e1002268. doi: 10.1371/journal.ppat.1002268 21966274 PMC3178577

[74] McKechnie JL Beltrán D Ferreira A-MM Vergara R Saenz L Vergara O. Mass cytometry analysis of the NK cell receptor–ligand repertoire reveals unique differences between dengue-infected children and adults. ImmunoHorizons (2020) 4(10):634–47. doi: 10.4049/immunohorizons.2000074 PMC8608029 33067399

[75] Pohlmeyer Christopher W Gonzalez Veronica D Irrinki A Ramirez Ricardo N Li L Mulato A. Identification of NK cell subpopulations that differentiate HIV-infected subject cohorts with diverse levels of virus control. J Virol (2019) 93(7):e01790-18. doi: 10.1128/jvi.01790-18 30700608 PMC6430550

[76] Mavilio D Benjamin J Daucher M Lombardo G Kottilil S Planta MA. Natural killer cells in HIV-1 infection: dichotomous effects of viremia on inhibitory and activating receptors and their functional correlates. Proc Natl Acad Sci USA (2003) 100(25):15011–6. doi: 10.1073/pnas.2336091100 PMC299884 14645713

[77] Vendrame E Seiler C Ranganath T Zhao NQ Vergara R Alary M. TIGIT is upregulated by HIV-1 infection and marks a highly functional adaptive and mature subset of natural killer cells. AIDS (2020) 34(6):801–13. doi: 10.1097/QAD.0000000000002488 PMC7148170 32028328

[78] Bauer S Groh V Wu J Steinle A Phillips JH Lanier LL. Activation of NK cells and T cells by NKG2D, a receptor for stress-inducible MICA. Science (1999) 285(5428):727–9. doi: 10.1126/science.285.5428.727 10426993

[79] Lee MJ Blish CA. Defining the role of natural killer cells in COVID-19. Nat Immunol (2023) 24(10):1628–38. doi: 10.1038/s41590-023-01560-8 PMC10538371 37460639

[80] Cifaldi L Doria M Cotugno N Zicari S Cancrini C Palma P. DNAM-1 activating receptor and its ligands: how do viruses affect the NK cell-mediated immune surveillance during the various phases of infection? Int J Mol Sci (2019) 20(15):3715. doi: 10.3390/ijms20153715 31366013 PMC6695959

[81] Zheng M Gao Y Wang G Song G Liu S Sun D. Functional exhaustion of antiviral lymphocytes in COVID-19 patients. Cell Mol Immunol (2020) 17(5):533–5. doi: 10.1038/s41423-020-0402-2 PMC7091858 32203188

[82] Cong J. Metabolism of natural killer cells and other innate lymphoid cells. Front Immunol (2020) 11:1989. doi: 10.3389/fimmu.2020.01989 32983138 PMC7484708

[83] Mira JC Gentile LF Mathias BJ Efron PA Brakenridge SC Mohr AM. Sepsis pathophysiology, chronic critical illness, and persistent inflammation-immunosuppression and catabolism syndrome. Crit Care Med (2017) 45(2):253–62. doi: 10.1097/CCM.0000000000002074 PMC5243156 27632674

[84] Gabrilovich DI Nagaraj S. Myeloid-derived suppressor cells as regulators of the immune system. Nat Rev Immunol (2009) 9(3):162–74. doi: 10.1038/nri2506 PMC2828349 19197294

[85] Elkabets M Ribeiro VS Dinarello CA Ostrand-Rosenberg S Di Santo JP Apte RN. IL-1beta regulates a novel myeloid-derived suppressor cell subset that impairs NK cell development and function. Eur J Immunol (2010) 40(12):3347–57. doi: 10.1002/eji.201041037 PMC3373225 21110318

[86] Li H Han Y Guo Q Zhang M Cao X. Cancer-expanded myeloid-derived suppressor cells induce anergy of NK cells through membrane-bound TGF-beta 1. J Immunol (2009) 182(1):240–9. doi: 10.4049/jimmunol.182.1.240 19109155

