# Supplementary material for: Immunometabolic features of natural killer cells are associated with infection outcomes in critical illness
Source: Front Immunol. 2024 Feb 15;15:1334882. doi: 10.3389/fimmu.2024.1334882 (PMC10902670; doi:10.3389/fimmu.2024.1334882)
Supplement: Supplementary file 1 [file DataSheet_1.pdf]

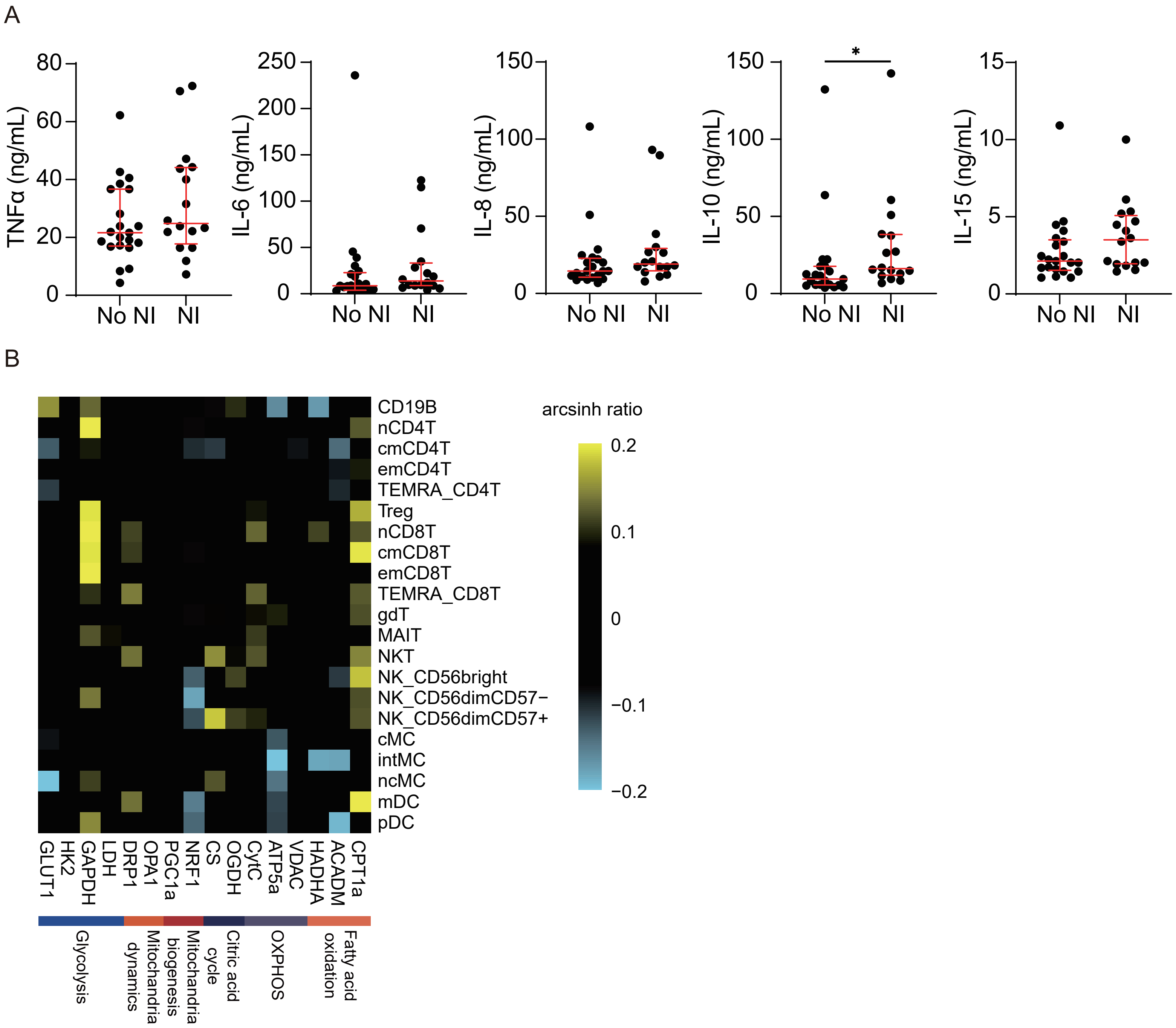

Supplementary Figure 1 (in relation to Figure 2). Serum cytokine levels and metabolic regulator profiles in different immune subsets in subgroup with nosocomial infection (NI) and that without NI. (**A**) Dot plots illustrate levels of circulating cytokines, including tumor necrosis factor (TNF) α, interleukin (IL)-6, IL-8, IL-10 and IL-15 in patients with NI (n=16) and those without NI (n=21). The line represents median and interquartile range, and *p* values are determined by Mann-Whitney U tests (* $p < 0.05$). (**B**) A heatmap is generated to depict the arcsinh ratio (NI/No NI) of all assessed metabolic regulators across immune subsets (please refer to the legend of **Figure 1** for the abbreviations).

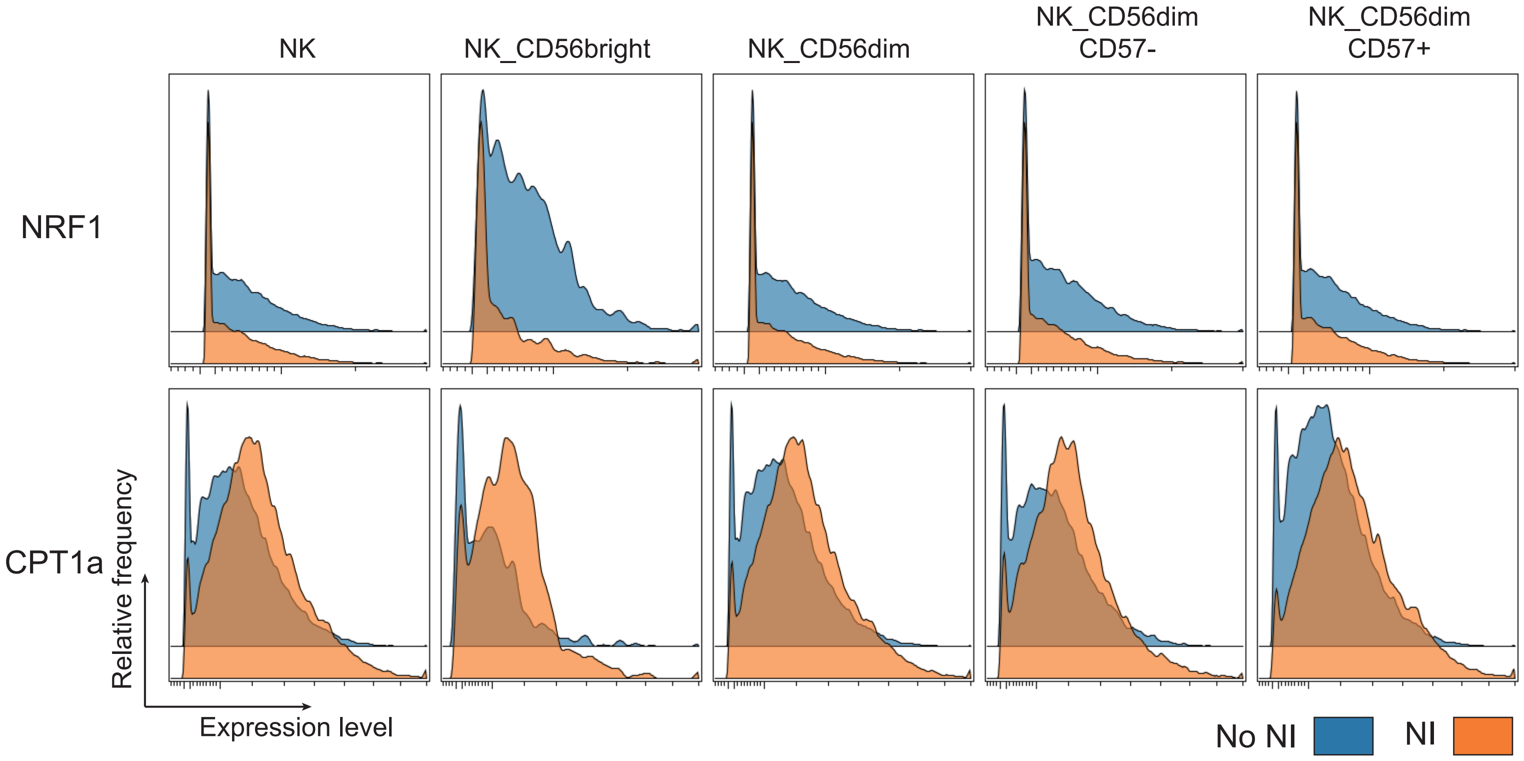

Supplementary Figure 2 (in relation to Figure 3). Expression of NRF1 and CPT1a in NK cell population. NRF1 and CPT1a expression levels are determined by CyTOF. The histogram plots represent the distribution of expression levels of NRF1 (upper) and CPT1a (lower) in the NK cells from patients with nosocomial infection (NI, in orange) and those without NI (in blue color).

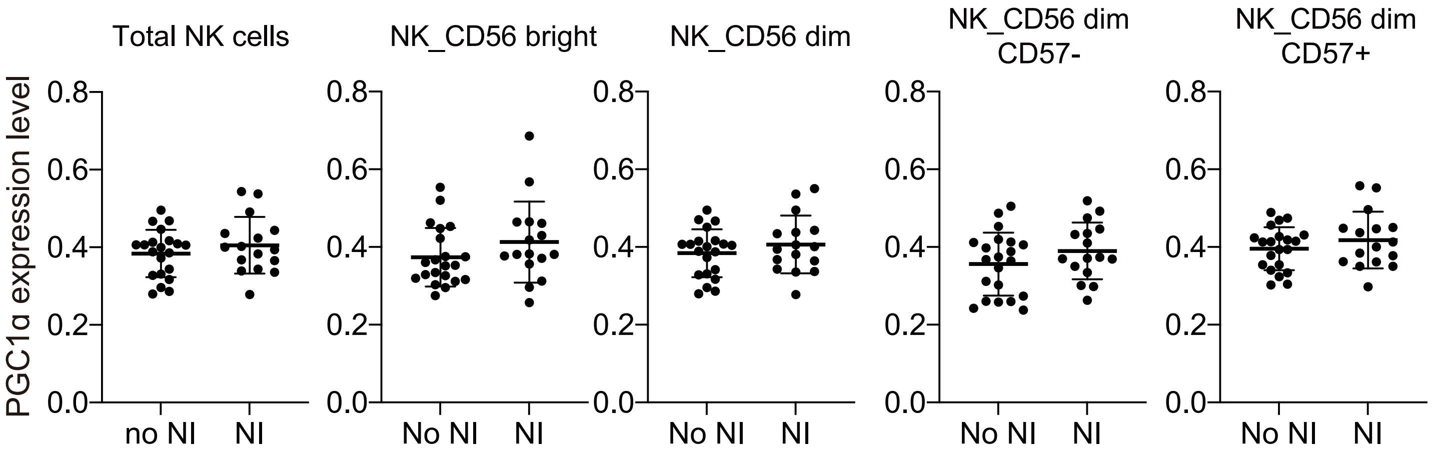

Supplementary Figure 3 (in relation to Figure 3). PGC1α expression in NK cells is not correlated with occurrence of nosocomial infection (NI) in the study population. The lines indicate mean ± standard deviation, and the comparison is performed using Mann-Whitney U tests.

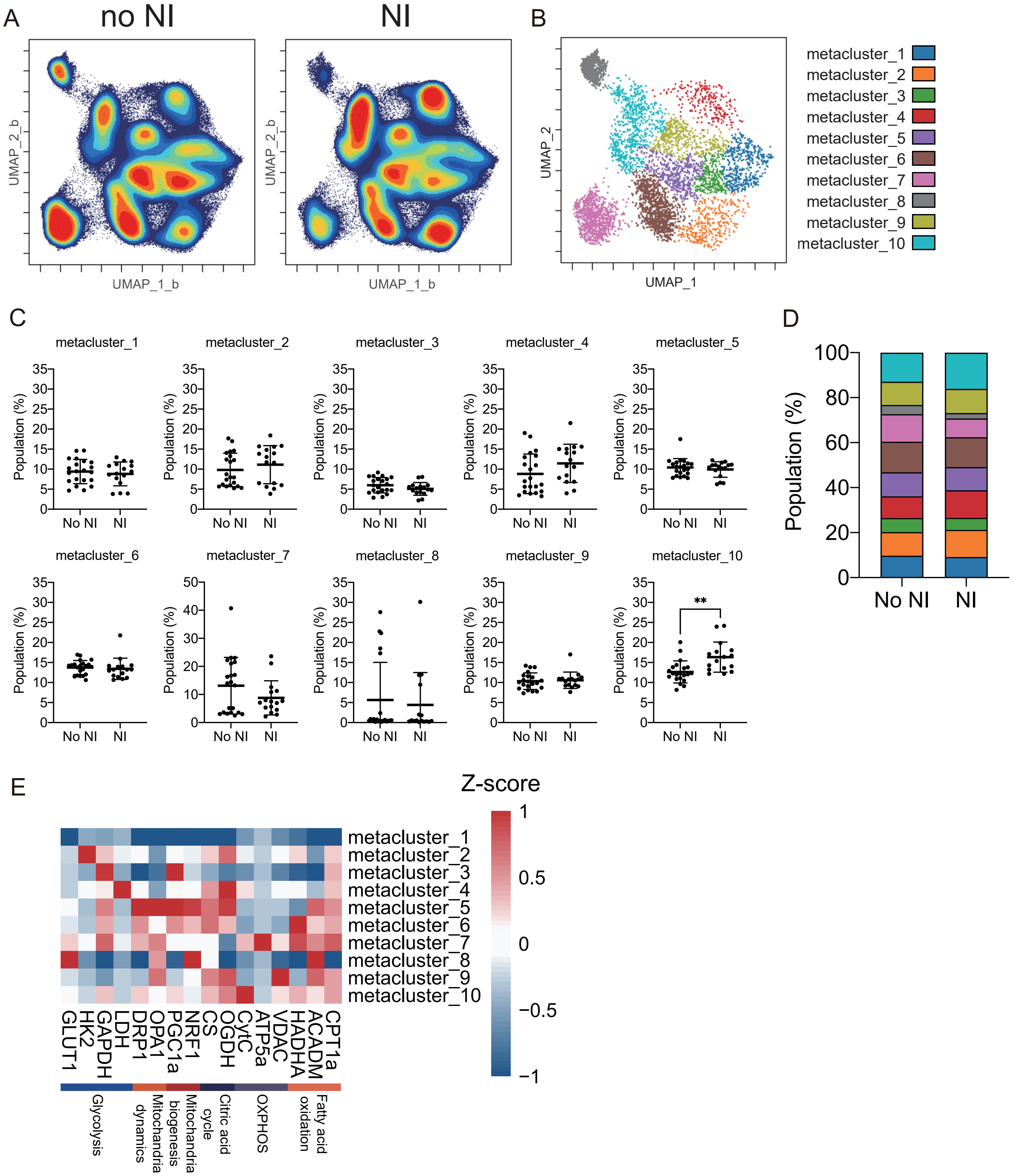

Supplementary Figure 4 (in relation to Figure 3). Metabolic profiles in NK cell population associated with nosocominal infection (NI) in the study population. (**A**) NK cells are subsampled for equal representation and concated from both NI and no NI patients. UMAP dimensionality reduction and visualization are performed based on metabolic regulators in the CyTOF panel. Data are represented as contour plots. (**B**) UMAP results are unsupervised clustered by FlowSOM analyses. Cell colors represent different metacluter. (**C**) Plots show the population (%) of each FlowSOM metacluster in subgroup with NI and that without. The lines represent the mean ± standard deviation, and *p* values are determined by Mann-Whitney U tests (** $p < 0.01$). (**D**) The bar graph summarizes the percentage of each FlowSOM metacluster in NI and non-NI subgroups. (**E**) A heatmap shows the normalized mean expression (z-score) of all assessed metabolic regulators across FlowSOM metaclusters (please refer to the legend of **Figure 1** for abbreviations).

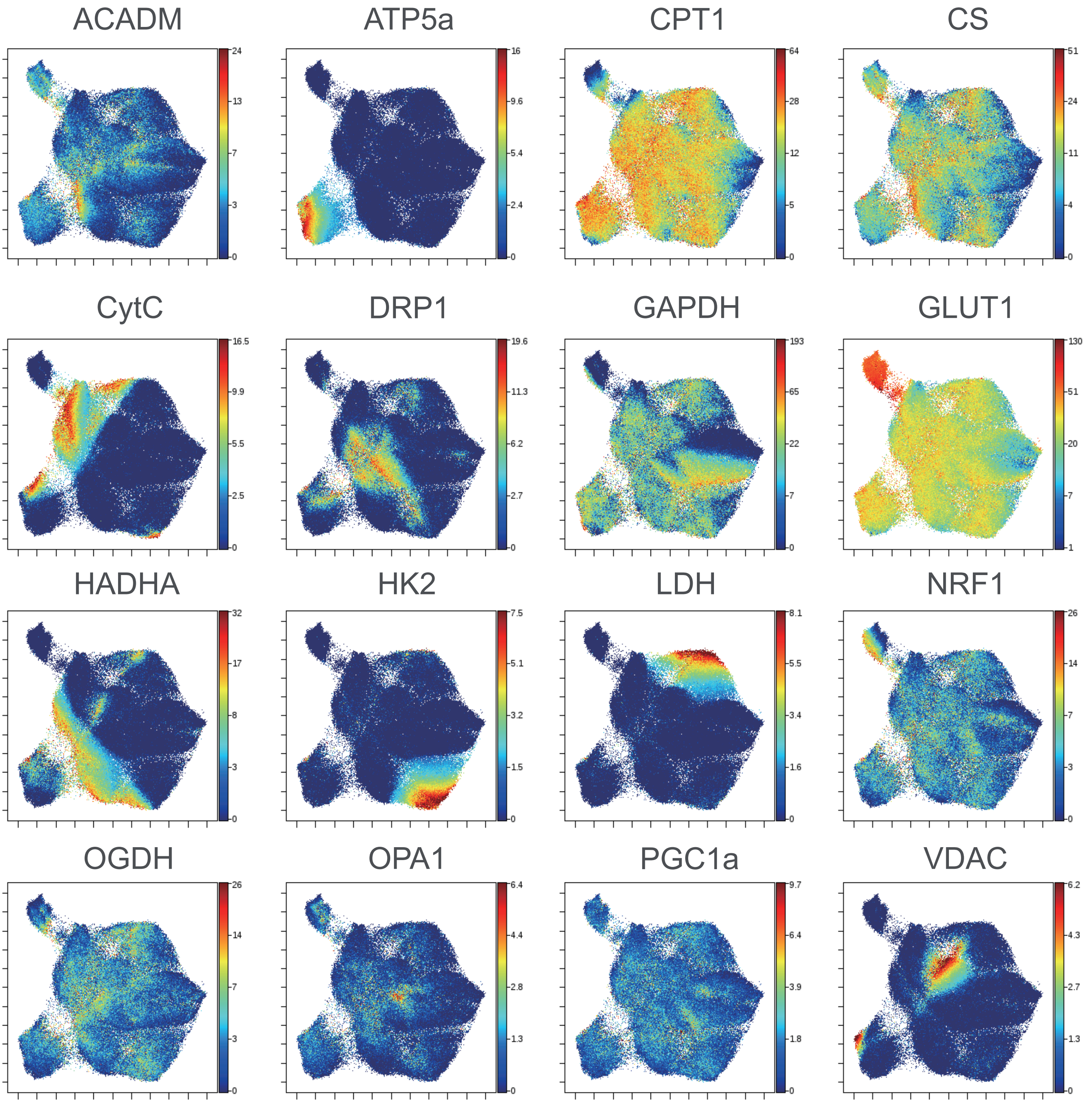

Supplementary Figure 5 (in relation to Figure 3 and Supplementary Figure 4). UMAP visualizations of median arcsinh-transformed intensities of metabolic regulators in the NK cell population (please refer to the legend of **Figure 1** for abbreviations).

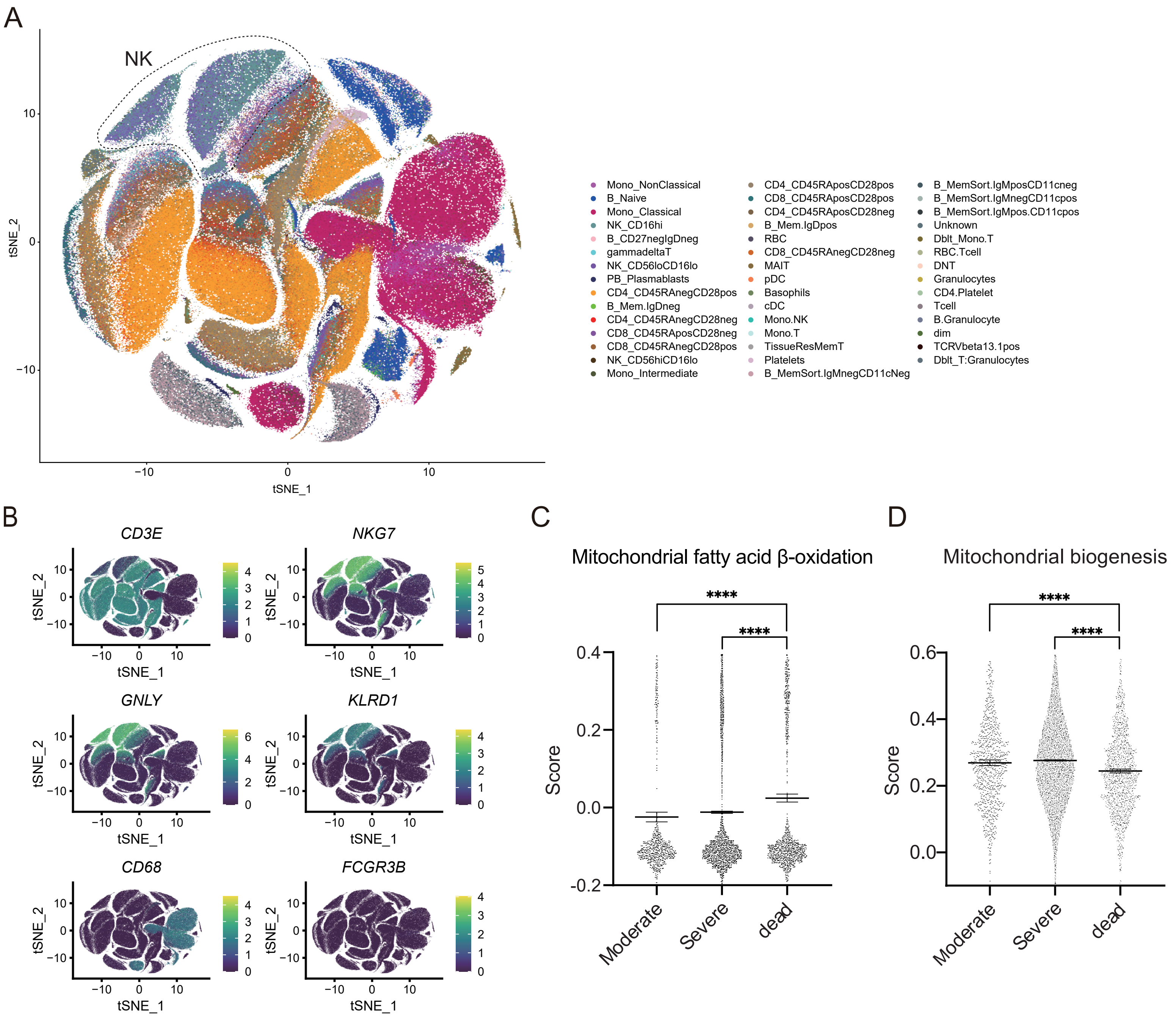

Supplementary Figure 6 (in relation to Figure 6). NK cell-specific immunometabolic features are associated with outcomes of critical COVID-19 infection (scRNA-seq analyses using GSE161918 data set). (**A**) A t-SNE plot showing clustering of major immune subsets. (**B**) The lineage specific marker genes for identifying NK cell population are shown. (**C** and **D**) The violin plots of z-scores for expression of genes in (**D**) mitochondrial fatty acid oxidation pathway and (**E**) mitochondrial biogenesis in subgroups categorized by disease seveirty and the survival outcome of critical COVID-19 infection. The lines denote mean ± standard deviation, and the *p* values are calculated using Kruskal-Wallis test and adjusted for multiple comparisons using the Dunn's method (**** $p < 0.0001$).
